# Pubic‐Related Radiographic Findings in Male Football Players With Long‐Standing Groin Pain, and Asymptomatic Controls — Are They Clinically Relevant?

**DOI:** 10.1111/sms.70068

**Published:** 2025-05-13

**Authors:** Mathias Fabricius Nielsen, Per Hölmich, Sonia Branci, Trine Torfing, Lasse Ishøi, Michael Bachmann Nielsen, Kristian Thorborg

**Affiliations:** ^1^ Sports Orthopedic Research Center – Copenhagen (SORC‐C), Department of Orthopedic Surgery Copenhagen University Hospital ‐ Amager‐Hvidovre Hvidovre Denmark; ^2^ Department of Diagnostic Radiology Rigshospitalet, Copenhagen University Hospital – Rigshospitalet Copenhagen Denmark

**Keywords:** athletes, football, groin pain, long‐standing pain, pubic symphysis, radiographs

## Abstract

The Aspetar pubic symphysis radiographic scoring protocol is reliable in male football players, but its clinical significance is unclear. We investigated the prevalence of pubic‐related radiographic findings and their association with groin pain and disability in male football players and asymptomatic controls. We included 39 symptomatic male football players with long‐standing groin pain, 18 asymptomatic male football players, and 20 asymptomatic male non‐football athletes. Standing anteroposterior pelvic radiographs were analyzed by two radiologists for pubic‐related bone lucency, proliferation, sclerosis, fragmentation, and joint space width (JSW, millimeters). Findings were combined into a Pubic Symphysis Radiographic Severity Score (PSRS Score, 0–8). Groin pain and disability were measured using the Five‐Second Squeeze Test (5SST, 0–10) and the Hip and Groin Outcome Score (HAGOS, 100–0). For symptomatic football players, asymptomatic football players, and asymptomatic non‐football athletes, the pubic‐related radiographic findings prevalence's were, respectively: bone lucency: 87%, 83%, and 40%; proliferation: 67%, 61%, and 25%; sclerosis: 64%, 50%, and 15%; and fragmentations: 15%, 6%, and 0%, while the mean JSW was 3 mm in all three groups. There were no differences between symptomatic and asymptomatic football players in any findings (*p* ≥ 0.39). Bone lucency, proliferation, and sclerosis were more frequent in football players than non‐football athletes (*p* < 0.002). PSRS Score showed poor correlation with 5SST and HAGOS. In conclusion, pubic‐related radiographic findings are not associated with groin pain or disability. Pubic‐related radiographic findings are more common in male football players than male non‐football athletes.

## 1 Introduction

Male athletes with long‐standing groin pain often undergo anteroposterior pelvic and lateral hip radiographs to screen for serious pathology and classify hip joint‐related groin pain based on bony morphology [1]. In addition to visualizing the hip joints, anteroposterior pelvic radiographs also display the pubic symphysis, which is considered a potential source of groin pain [2, 3]. In healthy adult males, the pubic symphysis typically has smooth, even, and well‐defined joint surfaces and a joint space width of 4–7 mm [4, 5, 6].

At least 13 studies have suggested that the pubic symphysis in male athletes with groin pain frequently displays radiographical abnormalities, such as joint space widening or narrowing, bone irregularities, erosions, cysts, sclerosis, bony proliferation and/or osteophyte formation (beaking), fragmentation, and accentuation of the gracilis origin [7, 8, 9, 10, 11, 12, 13, 14, 15, 16, 17]. However, the prevalence of joint space widening or narrowing, bone irregularities, erosions, sclerosis, and accentuation of the gracilis origin varies between 5% and 83% in athletes with groin pain [9, 10, 18, 19], and 0% to 85% in asymptomatic controls [9, 18, 20, 21, 22], while no radiographical abnormalities are seen in 17% to 44% of athletes with groin pain [9, 10, 18, 19]. These highly variable findings may be attributed to substantial population heterogeneity within and between studies, inclusion of inappropriate control groups, and methods with unclear definitions of pubic‐related radiographic findings [17, 20, 22]. Specifically, the prevalence of pubic‐related radiographic findings may also be influenced by increasing age and high‐impact load‐bearing sports (football, hockey, etc.) as compared with technical and lower impact/load sports (running, martial arts, etc.) [5, 23, 24]. This emphasizes the need for clear definitions of radiographical findings, robust study methodology, and controlling for age and sport when studying the associations between pubic‐related radiographic findings and groin pain.

The Aspetar pubic symphysis radiographic scoring protocol was recently developed to provide a detailed and reproducible scoring system for the pubic symphysis and adjacent bone with clear definitions of five main findings (bone lucency, proliferation, sclerosis, fragmentation, and joint space width) and ten subclassifications [22]. The protocol has moderate to good intra‐ and interrater reliability in healthy male football players [22], indicating an acceptable reliability for group‐level assessments and research. Serner et al. reported that pubic‐related radiographic findings were present in up to 65% of asymptomatic male football players [22], however, the prevalence in symptomatic football players with groin pain remains unknown. To understand the potential clinical relevance of radiographic findings scored using the Aspetar protocol, it is essential to first determine their associations with groin pain, pain intensity, and disability. Clarifying these associations might potentially benefit the diagnostic work‐up of athletes with long‐standing groin pain and improve the understanding of pathogenesis and mechanisms underlying groin pain.

The aim of this study was to investigate the associations between pubic‐related radiographic findings and pain in male athletes with long‐standing groin pain. The study had three specific objectives: (1) To compare pubic‐related radiographic findings, from the Aspetar protocol, in symptomatic male football players with groin pain with asymptomatic male football players and asymptomatic male non‐football athletes. (2) To explore whether the total number of pubic‐related radiographic findings is associated with groin pain, groin pain intensity, and disability. (3) To explore the influence of individual pubic‐related radiographic findings on groin pain intensity and disability in symptomatic football players.

## 2 Methods

### 2.1 Design

This is a cross‐sectional case–control study based on retrospective clinical data and radiographic images collected in 2011–2012 as part of a larger epidemiological project on hip and/or groin pain (ethical reference: H‐2‐2010‐127, data reference: 2011‐41‐5964) [24, 25, 26]. The epidemiological project was conducted in an orthopedic and a radiological outpatient clinic at a public university hospital in the Capital Region of Denmark. Participants provided written informed consent when included in the original project. In this study, new data were generated using the Aspetar pubic symphysis radiographic scoring protocol to reevaluate X‐ray images. This study was approved by the Danish National Committee on Health Research Ethics (NVK 2117722) and the Capital Region Data agency (P‐2021‐497).

### 2.2 Participants

**FIGURE 1:**
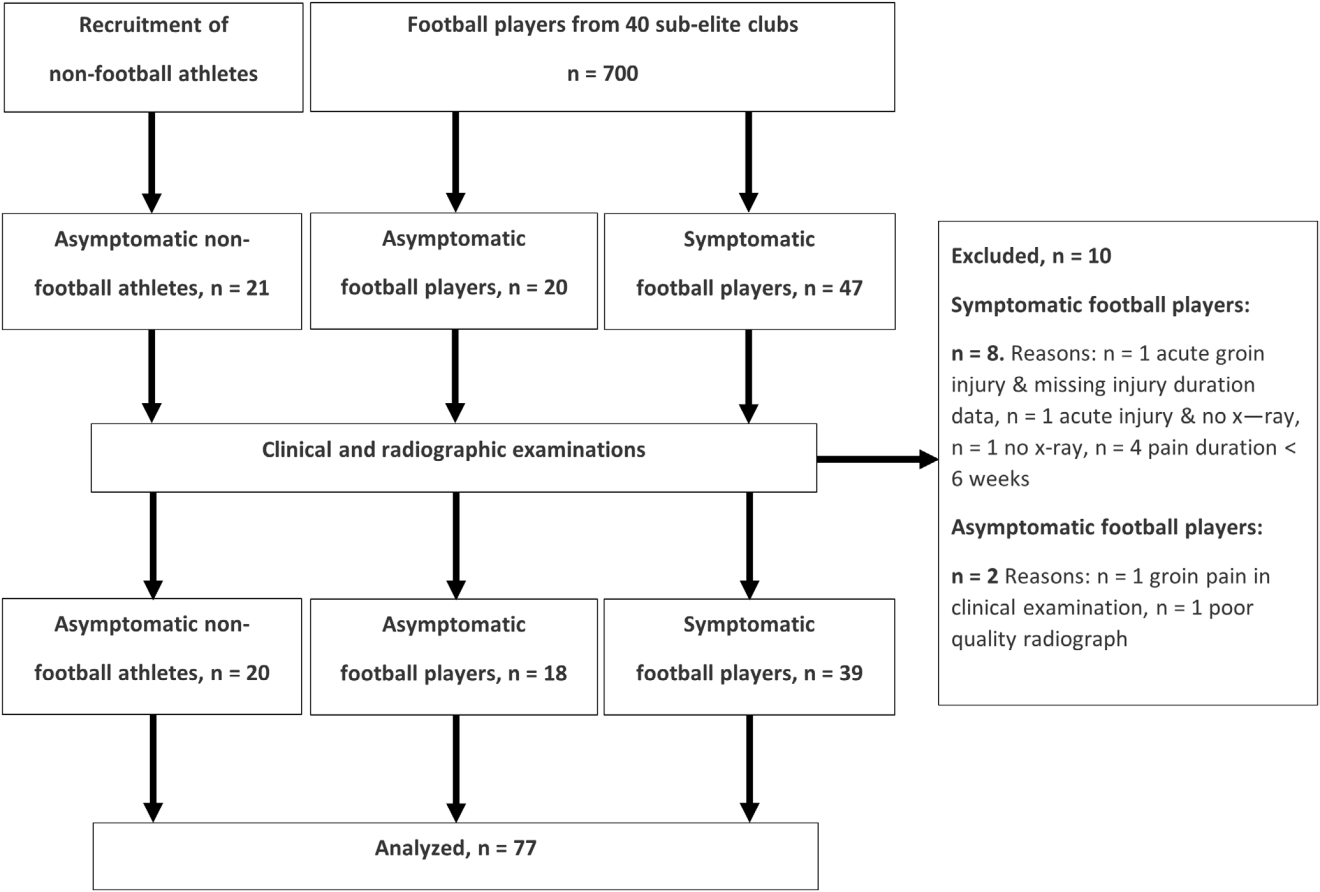
Participant flow.

Symptomatic and asymptomatic football players were recruited from a pool of 700 male senior players (18+ years) from 40 sub‐elite football clubs (tier 2–5) in Denmark. Asymptomatic non‐football athletes were recruited from sports clubs, educational institutions, and fitness centres. The symptomatic and asymptomatic participants were matched by age, sport, and athletic exposure to isolate the effect of sport and load as a potential confounder in the association between radiographic findings and groin pain. The participant flow is presented in Figure 1.

Symptomatic football players were male, ≥ 18 years old, participated in 2–4 weekly football sessions, and had self‐reported groin pain during football or running with a duration of ≥ 6 weeks prior to the clinical examination. Asymptomatic football players met the same criteria but had no hip or groin pain in the past year. Asymptomatic non‐football athletes were male, ≥ 18 years old, participated in 2–4 weekly training sessions, had no hip or groin pain in the past year, had not played football regularly in the past 10 years, and did not practice sports associated with groin pain (handball, rugby and ice hockey). Exclusion criteria for all groups were systemic disorders, obesity, depression, alcohol or drug abuse, and other conditions that may cause groin pain.

### 2.3 Data Collection

From the original project, this study included data from a baseline questionnaire, the Copenhagen Five‐Second‐Squeeze Test (5SST), the Copenhagen Hip and Groin Outcome Score (HAGOS), a standardized clinical examination and one anteroposterior pelvic view radiograph, and a cross‐table lateral view radiograph of each hip (see Supplementary File For Details).

#### 2.3.1 Radiographic Examination

All radiographs were accessed in Agfa IMPAX Client (Bundle 5) (Agfa‐Gevaert Group, Belgium), and evaluated individually by two musculoskeletal radiologists, SB and TT, with 11 and 25 years of experience, respectively. They rated pubic‐ and hip‐related radiographic findings using the Aspetar pubic symphysis radiographic scoring protocol, and commonly used methods for assessment of hip‐related radiographic findings (see Supplementary File For Details). Following individual ratings, consensus meetings were held to resolve any disagreements and to establish a dataset of consensus‐based ratings. This consensus dataset was used for all analyses in this study.

**FIGURE 2:**
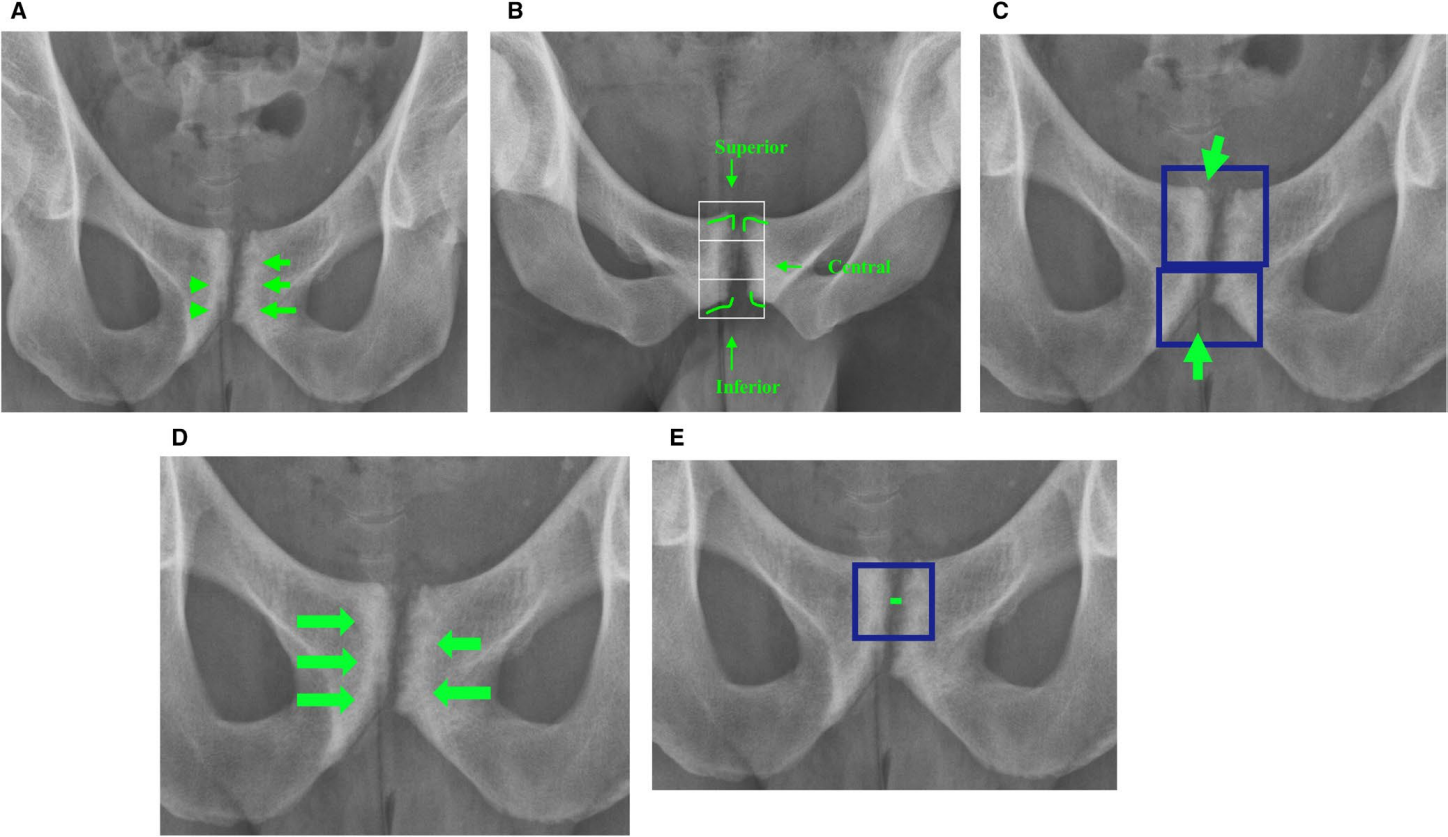
Pubic‐related radiographic findings as described by Serner et al. [22].^.^ (A) Bone lucency. Definition: “A clear area of decreased attenuation compared to the surrounding bone, which corresponds to an erosion‐like configuration and/or cyst.” [22], Subclassifications: *Erosion‐Like Configuration (ELC), Superior/Central ELC, Inferior ELC and Cysts*. Definitions: *ELC*: “Irregularities of the cortical bone surface, potentially accompanied by loss of the adjacent trabecular bone.” [22], *Superior/Central ELC*: ELC at the superior two thirds of the joint surface [22], *Inferior ELC*: ELC at the lower third of the joint surface ‐ “If the entire lower half was considered to have an erosion‐like configuration, both of the above were scored as positive” [22], *Cysts*: “Areas of bone lucency with a sclerotic rim inside the trabecular bone compartment, without accompanying cortical bone surface irregularity.” (B) Proliferation. Definition: “Clear osteophyte outgrowths at the joint margins or within the articular space.” [22], Subclassifications: *Superior, Central and Inferior Proliferation*. Definitions: *Superior proliferation*: “This can be considered “bone spurs” or classified as “pubic beaking” when bilateral. Well rounded (smooth) bumps at the superior aspect, even if asymmetrical in size, were not considered proliferation. For superior proliferation, the “sharpness” of the superior bone corner angle was used for assistance with angles higher than 90 deg. (obtuse angle) considered “rounded” and scored negative/absent, whereas angles lower than 90 deg. (acute angle) were considered “sharp” and scored as positive/present.” [22], *Central proliferation*: “Proliferation at the central portion of the articular space.” [22], *Inferior proliferation*: “Similar considerations as superior proliferation.” [22]. (C) Fragmentation. Definition: “Clear loose fragment(s) within the symphyseal joint space, or at the inferior medial margin of the pubic bone.” [22], Subclassifications: *Central and inferior fragmentation*. Definitions: *Central fragmentation*: “Clear loose fragment(s) within the symphyseal joint space” [22], *Inferior fragmentation*: “Clear loose fragment(s) at the inferior medial margin of the pubic bone.” [22]. (D) Sclerosis. Definition: “A clear area of increased attenuation of the subchondral bone compared to the surrounding bone, corresponding to an area of increased bone density.” [22]. (E) Pubic Symphysis Joint Space Width. Definition: “Symphyseal joint space measured in millimeters at the narrowest point of the joint surfaces.” [22], Subclassification: *Narrow Joint Space Width*. Definition: *Narrow Joint Space Width*. “if measured to less than 3mm” [22].

Pubic‐related radiographic findings are illustrated and defined in Figure 2. Hip‐related radiographic findings are included as descriptive variables and illustrated and defined in the Supplementary File. Findings were rated absent or present for both sides (right and left), except for pubic joint space width, which was measured in millimeters (nearest whole number), and narrow joint space (< 3 mm) which was measured once for each joint. Findings needed to be unmistakably present to be rated present. For pubic‐related findings, the intra‐and interrater reliability ranged from slight (*κ* = 0.07, ICC 2,1 = 0.17) to almost perfect (*κ* = 0.86, ICC 2,1 = 0.78) [27].

#### 2.3.2 Pubic Symphysis Radiographic Severity Score

To assess the extent of pubic‐related findings, we combined the total number of dichotomous findings into a Pubic Symphysis Radiographic Severity Score (PSRS) for each participant. Four versions of the PSRS were developed to account for different levels of assessment: individual level, side level, main finding level, and subclassification level. All four versions of the PSRS Score are presented in Table 1. In the aggregated scores, equal weight was assigned to each finding. Consequently, narrow joint space was only included in the PSRS Scores at the individual level.

**TABLE 1:** Pubic symphysis radiographic severity (PSRS) score.

Version	Assessment level	Included findings	Score
**PSRS Score 1**	Each main finding assessed on a person level.	Bone lucency (0–1) Proliferation (0–1) Fragmentation (0–1) Sclerosis (0–1) Narrow joint space (0–1).	0–5
**PSRS Score 2**	Each main finding assessed on a side level, that is left and right in total.	Bone lucency (0–2) Proliferation (0–2) Fragmentation (0–2) Sclerosis (0–2)	0–8
**PSRS Score 3**	Each subclassification finding assessed on a person level.	Superior/central Erosion‐Like Configuration (0–1) Inferior Erosion‐Like Configuration (0–1) Cysts (0–1) Superior proliferation (0–1) Central proliferation (0–1) Inferior proliferation (0–1) Central fragmentation (0–1) Inferior fragmentation (0–1) Sclerosis (0–1) Narrow joint space (0–1)	0–10
**PSRS Score 4**	Each subclassification finding assessed on a side level, that is left and right in total.	Superior/central Erosion‐Like Configuration (0–2) Inferior Erosion‐Like Configuration (0–2) Cysts (0–2) Superior proliferation (0–2) Central proliferation (0–2) Inferior proliferation (0–2) Central fragmentation (0–2) Inferior fragmentation (0–2) Sclerosis (0–2)	0–18

#### 2.3.3 Baseline Questionnaire

Before examinations participants completed a baseline questionnaire collecting information on age (years), height (centimeters), body mass (kilograms), preferred kicking leg (right/left), number of weekly football or sport training sessions and weekly training hours. For symptomatic football players, the questionnaire also included current football/sports participation status (playing/not‐playing), duration of symptoms (months), symptom location (right/left/bilateral), and worst pain in the previous week, measured on a numeric pain rating scale (NRS) from 0, “no pain,” to 10, “worst pain imaginable”.

#### 2.3.4 The Copenhagen 5‐Second Squeeze Test

The 5SST was used to assess self‐reported groin‐related pain intensity on a numeric rating scale from 0 “no pain” to 10 “worst pain imaginable” [28]. The 5SST was performed with athletes lying supine, with 0° hip flexion and straight legs on an examination bench. The forearm of the assessor was placed between the ankles of the athlete as resistance against bilateral hip adduction, and the arm was placed approximately 5 cm proximal to the medial malleoli, resulting in approx. 10°–15° hip abduction angle bilaterally (See Supplementary file for image of setup). Participants were instructed to perform a 5 s maximum and continuous bilateral isometric hip adduction squeeze with gradual increase in force production if necessary. The instruction was “3–2‐1‐Go‐push‐push‐push‐push‐and‐stop”. This was followed by rating the intensity of evoked groin pain during or right after the test.

#### 2.3.5 The Copenhagen Hip and Groin Outcome Score

HAGOS was used to assess self‐reported groin‐related disability [29]. HAGOS includes 37 items on the subscales: pain, symptoms, physical function in daily living (ADL), physical function in sport and recreation (Sport), participation in physical activities (PA), and quality of life (QoL). Items are answered on a five‐point Likert scale from 0 to 4. Subscales are normalized into a summary score ranging from 0 (extreme symptoms) to 100 (no symptoms) [29]. HAGOS is valid, reliable, and responsive in Danish [29]. HAGOS was recently validated using modern test theory, which resulted in revised HAGOS subscale scores utilizing only 30 items [30]. The revised HAGOS scores were used in this study. The original HAGOS scores are presented in the Appendix Table A1 to allow comparison with studies using the original version.

#### 2.3.6 Clinical Examination

All participants underwent a standardized bilateral clinical examination of the hip and groin regions in accordance with the framework by Hölmich et al. [31], which included palpation, manual resistance, or stretching tests. Tests were considered positive if they elicited known and recognizable groin pain. If discomfort but not known groin pain was reported, the test was rated negative. The symptomatic football players were classified with clinical entities of groin pain according to the Doha Classification system [3], based on the positive pain provocation tests. This approach has acceptable reliability [32]. Pain provocation tests and clinical entities of groin pain are described and defined in the Tables S1 and S2.

### 2.4 Sample Size Considerations

A maximum of 88 participants underwent clinical and radiological examinations in the original project (H‐2‐2010‐127). Assuming all participants had complete datasets and all radiographs were of adequate quality, this would result in a fixed sample size of 47 symptomatic football players, 20 asymptomatic football players, and 21 asymptomatic non‐football athletes. Based on a *Χ* ^2^‐test, an alpha level of 0.05, and a power of 0.80, this would allow for detecting a statistically significant difference of 34% between groups, corresponding to a moderate to large effect size (Cohens h = 0.75). Using the R package “pwr, we estimated that a fixed number of 48 symptomatic football players, an alpha level of 0.05, and a power of 0.80 would allow us to detect statistically significant correlation coefficients of at least 0.39.

### 2.5 Statistical Analyses

Statistical analyses were performed in R version 4.3.0, RStudio 2022.12.0 + 353. Dichotomous pubic‐related findings were analyzed and presented as categorical data. Pubic joint space width (mm), the 5SST, the HAGOS subscales, and the PSRS Scores were treated and presented as continuous variables.

Differences in pubic‐related radiographic findings between groups were examined using *Χ* ^2^‐tests and the “tableone” R‐package. For pair‐wise comparison between groups, odds ratios with 95% confidence intervals were estimated for each pubic‐related radiographic finding using the “epitools” and “effectsize” R‐packages. Differences in pubic joint space width and PSRS Scores between groups were investigated using the “multcomp” R‐package to conduct a one‐way analysis of variance (ANOVA) with a post hoc Tukey's test. For the primary between‐group comparisons, pubic‐related findings on an individual level, and not on the side‐specific level, were used. However, a series of sensitivity analyses comparing only right‐sided findings and left‐sided findings between groups were also conducted, and only findings from sides with groin pain from symptomatic football players were analyzed.

For symptomatic football players, we explored the associations between PSRS Scores, 5SST, and HAGOS subscales using the “ggplot2” R‐package to create scatter plots with Locally Estimated Scatterplot Smoothing (LOESS). We also used the “correlation” R‐package to estimate Spearman's correlation coefficients with 95% confidence intervals. Correlation coefficients were interpreted as negligible (0.0–0.10), weak (0.10–0.39), moderate (0.40–0.69), strong (0.70–0.89), or very strong (0.90–1.00).

To explore the influence of the pubic‐related radiographic findings on 5SST and HAGOS subscales in symptomatic football players, we constructed seven multivariate linear regression models using the “stats” R‐package. In each model, the dependent variable was either the 5SST or a HAGOS subscale, and the independent variables were the five main pubic‐related radiographic findings (bone lucency, proliferation, fragmentation, sclerosis, narrow joint space) at the individual level.

Conclusions from statistical analyses are based on unadjusted results to preserve the study's exploratory nature and avoid overlooking findings relevant for future research. However, as Type‐1 error (false positives) was highlighted as a key limitation during peer review, we performed post hoc *p* value adjustments using the Benjamini–Hochberg approach [33]. The adjusted *p* values are included in Tables and the Appendix.

## 3 Results

### 3.1 Participants

Of 88 participants with available data, we included 39 symptomatic football players with long‐standing groin pain, 18 asymptomatic football players, and 20 asymptomatic non‐football athletes (Figure 1). Asymptomatic non‐football athletes participated in fitness (*n* = 8), running (*n* = 5), martial arts (*n* = 3), cycling (*n* = 1), parkour (*n* = 1), kayak (*n* = 1), and basketball (*n* = 1). Descriptive data are in Table 2.

**TABLE 2:** Descriptive data.

		Symptomatic football players		Asymptomatic football players		Asymptomatic non‐football athletes	
***n***		39		18		20	
**Age**, years, mean (SD)		24.5 (3.2)		23.1 (2.7)		25.9 (5.9)	
**Height**, cm, mean (SD)		181.9 (5.7)		181.0 (4.6)		181.9 (5.8)	
**Weight**, kg, mean (SD)		77.9 (6.6)		79.1 (6.0)		80.3 (8.7)	
**Body Mass Index**, mean (SD)		23.5 (1.4)		24.2 (1.5)		24.3 (2.8)	
**Training pr week**, hours, mean (SD)		5.6 (1.6)		5.6 (1.0)		7.5 (2.9)	
**Training pr week**, sessions, median (IQR)		4 (3–4)		4 (3–4)		3 (3–3)	
**Kicking Leg**, right *n* (%)		29 (74%)		13 (81%)		18 (95%)	
**Symptomatic side**	*Right*, *n* *(%)*	16 (41%)		—		—	
	*Left*, *n* (%)	7 (18%)		—		—	
	*Bilateral*, *n* (%)	14 (36%)		—		—	
**Long‐standing groin pain**, *n* (%)		39 (100%)		—		—	
**Pain duration**, months, median (IQR)		10 (5–36)					
**Injury mechanism**	*Acute*, *n* (%)	15 (39%)		—		—	
	*Gradual*, *n* (%)	24 (2%)		—		—	
**Pain in preceding week**, 0–10, median (IQR)		8 (6–9)		0 (0–0)		0 (0–0)	
**5SST**, 0–10, median (IQR)		5 (4–8)		0 (0–0)		0 (0–0)	
**HAGOS**, Revised							
*Pain*, median (IQR)		84 (67–91)		100 (97–100)		100 (100–100)	
*Symptoms*, median (IQR)		62 (50–73)		92 (83–100)		96 (91–100)	
*ADL*, median (IQR)		81 (62–87)		100 (100–100)		100 (100–100)	
*Sport*, median (IQR)		46 (33–62)		100 (92–100)		100 (100–100)	
*PA*, median (IQR)		37 (19–62)		100 (100–100)		100 (100–100)	
*QoL*, median (IQR)		44 (28–56)		100 (94–100)		100 (100–100)	
**Clinical entities of groin pain**		**Right**	**Left**				
Adductor‐related groin pain, *n* (%)		19 (49%)	18 (46%)	—		—	
Iliopsoas‐related groin pain, *n* (%)		20 (51%)	23 (59%)	—		—	
Inguinal‐related groin pain, *n* (%)		18 (46%)	18 (46%)	—		—	
Pubic‐related groin pain, *n* (%)		5 (13%)		—		—	
Total entities, median (IQR)		3 (2–4)		—		—	
**Hip‐related Radiographic Findings**		**Right**	**Left**	**Right**	**Left**	**Right**	**Left**
Alpha Angle, mean (SD)		53 (13)	55 (11)	52 (13)	52 (12)	48 (10)	49 (11)
*Cam morphology*, *n* (%)		9 (23%)	10 (26%)	3 (17%)	5 (28%)	2 (10%)	3 (15%)
Lateral Center Edge Angle, mean (SD)		30 (5)	30 (5)	27 (7)	28 (9)	30 (6)	31 (5)
Acetabular Index Angle, mean (SD)		7 (4)	6 (5)	9 (4)	9 (5)	7 (4)	5 (4)
*Pincer Morphology*, *n* (%)		2 (8%)	1 (4%)	1 (10%)	1 (10%)	1 (7%)	0 (0%)
*Borderline Hip Dysplasia*, *n* (%)		5 (21%)	5 (21%)	6 (60%)	3 (33%)	5 (33%)	1 (7%)
*Hip Dysplasia*, *n* (%)		1 (4%)	1 (4%)	2 (20%)	3 (30%)	1 (7%)	0 (0%)
Hip joint space width, mm, mean (SD)		5 (1)	5 (1)	5 (1)	5 (1)	5 (1)	5 (1)
Cross‐Over sign, *n* (%)		12 (50%)	14 (58%)	4 (40%)	6 (60%)	7 (47%)	7 (47%)
Ischial Spine Sign, *n* (%)		15 (63%)	14 (58%)	4 (40%)	6 (60%)	8 (53%)	9 (60%)
Posterior Wall Sign, *n* (%)		17 (71%)	19 (79%)	5 (50%)	6 (60%)	9 (60%)	8 (53%)

*Note:* Missing/censored data: Asymptomatic football players, *n* = 1, Reason: no clinical examination, 5SST or HAGOS, *N* for the Hip‐related Radiographic Findings: for anteroposterior images: symptomatic football players *n* = 24, asymptomatic football *n* = 10, asymptomatic non‐football *n* = 15. Cam morphology: alpha angle > 60°. Pincer morphology: Lateral Center Edge Angle ≥ 40° OR (Lateral Center Edge Angle ≥ 35° AND Acetabular Index Angle < 0°). Borderline Hip Dysplasia: Lateral Center Edge Angle = 20° to 25°. Hip dysplasia: Lateral Center Edge Angle < 20° OR Acetabular Index Angle > 13°.

### 3.2 Pubic‐Related Radiographic Findings

**FIGURE 3:**
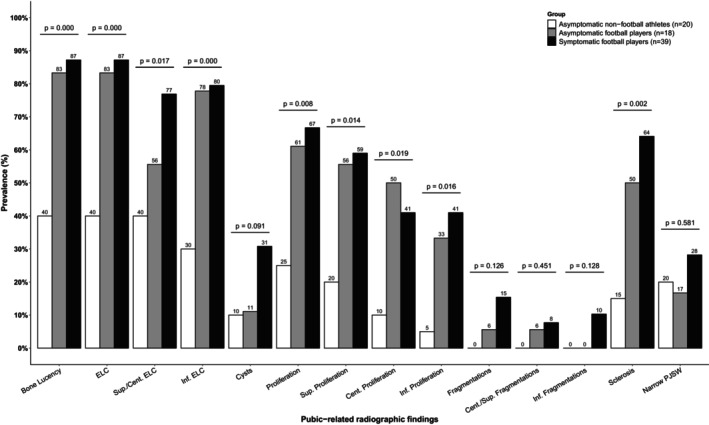
Prevalence of pubic‐related radiographic findings. *p* values are from *χ* ^2^‐tests across all three groups. ELC = Erosion‐Like Configuration.

**FIGURE 4:**
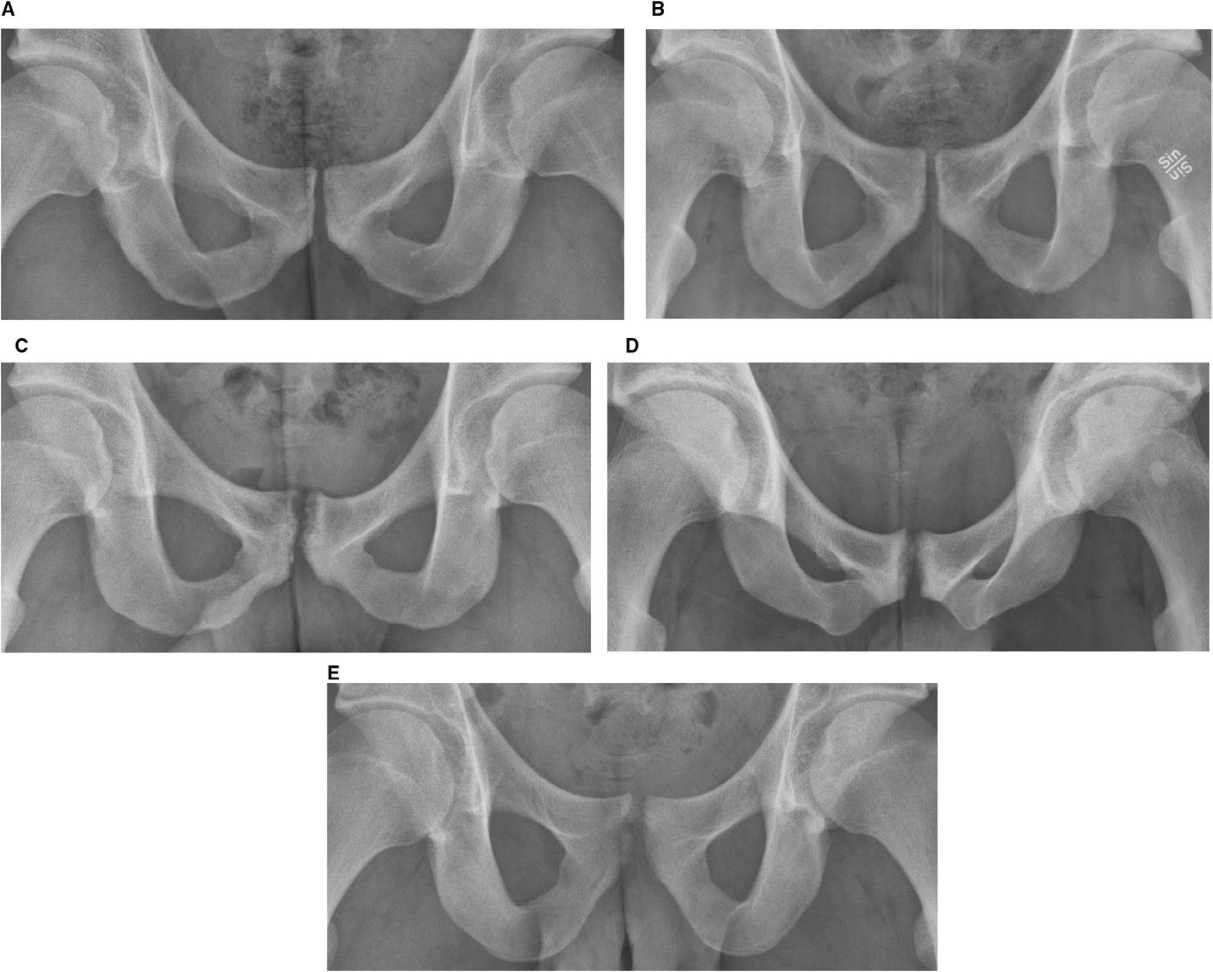
Examples of anteroposterior radiographs of athletes with and without groin pain. (A) Symptomatic football player: 26 years, 178 cm, 72 kg, dominant leg: Right, bilateral groin pain, pain duration: 2 months, worst pain last week 8, 5SST: 8, HAGOS Pain 100, Symptoms 66.7, ADL 81.3, Sport 37.5, PA 25, QoL 75; Clinical Entities: Bilateral adductor‐related, bilateral iliopsoas‐related, bilateral inguinal‐related, and pubic‐related groin pain. Pubic‐related radiographic findings total = 0 and JSW: 3 mm. (B) Symptomatic football player: 25 years, 182 cm, 75 kg, dominant leg: Right, right‐sided groin pain, pain duration: 6 weeks, worst pain last week: 7, 5SST: 8, HAGOS Pain 78.1, Symptoms 54.2, ADL 68.8, Sport 42.7, PA 0.0, QoL 43.8; Clinical Entities: Right‐sided adductor‐related groin pain, right‐sided inguinal‐related groin pain. Pubic‐related radiographic findings total = 0 and JSW: 4 mm. (C) Asymptomatic football player: 23 years, 180 cm, 79 kg, dominant leg: Right, HAGOS Pain 100, Symptoms 100, ADL 100, Sport 100, PA 100, QoL 100, Pubic‐related radiographic findings total = 14: Bilateral bone lucency, ELC, central/superior ELC; right‐sided cysts, bilateral proliferation, superior proliferation, central proliferation, inferior proliferation; bilateral sclerosis; JSW = 2 mm. (D) Symptomatic football player: 24 years, 188 cm, 80 kg, dominant leg: Right, bilateral groin pain, pain duration: 48 months, worst pain last week: 9, 5SST: 6, HAGOS Pain 37.5, Symptoms 29.2, ADL 31.3, Sport 29.2, PA 12.5, QoL 18.8; Clinical Entities: Bilateral adductor‐related, bilateral iliopsoas‐related, left inguinal‐related, and pubic‐related groin pain; Pubic‐Related Radiographic Finding total = 13: Bilateral findings of Bone lucency, ELC, central/superior ELC, proliferation, superior proliferation, central proliferation, and sclerosis. Left sided inferior proliferation, fragmentation, inferior fragmentation. JSW 1 mm. (E) Asymptomatic non‐football athlete: 19 years, 184 cm, 72 kg, dominant leg: Left, HAGOS Pain 100, Symptoms 100, ADL 100, Sport 100, PA 100, QoL 100, Pubic‐related radiographic findings: Bilateral bone lucency, ELC, central/superior ELC; left‐sided cysts, bilateral proliferation, superior proliferation; right‐sided central proliferation, and JSW = 2 mm.

No significant differences were observed in pubic‐related findings between symptomatic and asymptomatic football players (Table 3, Figure 3, and Appendix Table A9). The prevalence of cysts, fragmentation (including subclassifications), and a narrow pubic joint space did not differ across symptomatic football players, asymptomatic football players, and non‐football athletes (Figure 3, Appendix Table A9). However, the prevalence of bone lucency, ELC (including subclassifications), proliferation (including subclassifications), and sclerosis differed significantly across all three groups (Figure 3, Appendix Table A9). To illustrate these findings, five different radiographs are visualized and described in Figure 4. Similar results were observed when symptomatic sides (right *n* = 32, left *n* = 23) were compared to asymptomatic athletes (Appendix Table A2).

**TABLE 3:** Pairwise comparison of pubic‐related findings on person level.

	Symptomatic football (*n* = 39) vs. Asymptomatic football (*n* = 18)				Symptomatic football (*n* = 39) vs. Asymptomatic non‐football (*n* = 20)				Asymptomatic football (*n* = 18) vs. Asymptomatic non‐football (*n* = 20)			
Pubic‐related findings	OR	(95% CI)	*p*	Adj. *p*	OR	(95% CI)	*p*	Adj. *p*	OR	(95% CI)	*p*	Adj. *p*
**Bone Lucency**	0.74	(0.12–5.38)	0.70	0.85	0.10	(0.02–0.42)	**< 0.00**	**< 0.00**	0.14	(0.02–0.73)	**0.01**	**0.04**
*Erosion‐Like Configuration*	0.74	(0.12–5.38)	0.70	0.85	0.10	(0.02–0.42)	**< 0.00**	**< 0.00**	0.14	(0.02–0.73)	**0.01**	**0.04**
Superior/Central ELC	0.38	(0.10–1.47)	0.13	0.29	0.21	(0.05–0.74)	**0.01**	**0.03**	0.54	(0.12–2.31)	0.52	0.61
Inferior ELC	0.90	(0.20–4.81)	1.00	1.00	0.12	(0.03–0.44)	**< 0.00**	**< 0.00**	0.13	(0.02–0.64)	**< 0.00**	**0.04**
*Cysts*	0.29	(0.03–1.55)	0.19	0.40	0.26	(0.02–1.36)	0.11	0.28	0.89	(0.06–13.64)	1.00	1.00
**Proliferation**	0.79	(0.22–3.00)	0.77	0.86	0.17	(0.04–0.64)	**0.01**	**0.02**	0.22	(0.04–1.02)	0.05	0.08
*Superior Proliferation*	0.87	(0.25–3.16)	1.00	1.00	0.18	(0.04–0.69)	**0.01**	**0.02**	0.21	(0.04–1.01)	**0.04**	0.08
*Central Proliferation*	1.43	(0.40–5.11)	0.58	0.77	0.16	(0.02–0.85)	**0.02**	0.05	0.12	(0.01–0.74)	**0.01**	**0.04**
*Inferior Proliferation*	0.72	(0.18–2.63)	0.77	0.86	0.08	(0.00–0.60)	**0.01**	**0.02**	0.11	(0.00–1.09)	**0.04**	0.08
**Fragmentations**	0.33	(0.01–3.06)	0.41	0.68	0.00	(0.00–1.58)	0.09	0.24	0.00	(0.00–35.10)	0.47	0.61
*Central Fragmentation*	0.71	(0.01–9.61)	1.00	1.00	0.00	(0.00–4.73)	0.54	0.77	0.00	(0.00–35.10)	0.47	0.61
*Inferior Fragmentation*	0.00	(0.00–3.27)	0.30	0.55	0.00	(0.00–2.93)	0.29	0.55	—	—	—	—
**Sclerosis**	0.57	(0.16–2.03)	0.39	0.68	**0.10**	(0.02–0.44)	**< 0.00**	**< 0.00**	0.19	(0.03–0.99)	**0.04**	0.08
**Narrow Pubic Joint Space**	0.51	(0.08–2.38)	0.51	0.77	0.64	(0.13–2.65)	0.55	0.77	1.24	(0.18–9.94)	1.00	1.00
**Pubic Joint Space**, mean diff.	−0.3	(−1.0 to 0.5)	0.40	0.61	0.1	(−0.5 to 0.7)	0.75	0.75	0.3	(−1.0 to 0.4)	0.26	0.61

*Note:* Interpretation: A lower odds ratio indicates a higher risk of findings in the symptomatic group. Bold *p*‐values are statistically significant.

Abbreviations: CI, confidence interval; OR, odds ratio.

### 3.3 Pubic Symphysis Radiographic Severity Scores

No significant differences were observed in any of the four PSRS Scores between symptomatic and asymptomatic football players (Appendix Table A3). However, all four PSRS Scores were significantly higher in the symptomatic football players compared with asymptomatic non‐football athletes (Appendix Table A3).

In symptomatic football players (*n* = 39), the PSRS Score 1 (0–5) correlated significantly to HAGOS Pain (rho = −0.40, 95% CI: −0.64 to −0.09, *p* = 0.01, adj. *p* = 0.08, Appendix Figure A5) and HAGOS QoL (rho = −0.34, 95% CI: −0.60 to −0.01, *p* = 0.04, adj. *p* = 0.13, Appendix Figure A13), while the PSRS Score 2 (0–8) correlated significantly to HAGOS Pain (rho = −0.39, 95% CI: −0.63 to −0.07, *p* = 0.01, adj. p = 0.08, Appendix Figure A6). All other correlations between PSRS Scores and 5SST and HAGOS subscales were negligible (*ρ* = 0.0*)* to weak (*ρ* ≤ −028.) and non‐significant (*p* values = 0.08 to 0.75, Appendix Table A4 and Appendix Figures A1–A16).

### 3.4 Influence of the Pubic‐Related Radiographic Findings on 5SST and HAGOS

In the symptomatic football players (*n* = 39), linear regression models for 5SST showed that bone lucency (β = −3.9, 95% CI: −7.0 to −0.8, *p* = 0.02) and narrow pubic joint space (β = −2.2, 95% CI: −4.2 to −0.2, *p* = 0.04) were associated with lower 5SST values, indicating lower groin pain intensity (Appendix Table A5).

In the models for HAGOS subscales, sclerosis was associated with lower HAGOS Pain (β = −20.8, 95% CI: −33.7 to −7.9, *p* < 0.00) and HAGOS ADL values (β = −20.6, 95% CI: −38.2 to −2.9, *p* = 0.03), indicating worse pain and impaired physical function in daily living (Appendix Table A6).

## 4 Discussion

This is the first study to use the Aspetar pubic symphysis radiographic scoring protocol for evaluating the pubic symphysis joint in male football players with long‐standing groin pain and asymptomatic controls. We found that the pubic‐related findings had similar prevalence in symptomatic football players and asymptomatic football players. Most pubic‐related findings (e.g., bone lucency, proliferation, and sclerosis) were more prevalent in male football players than in asymptomatic non‐football athletes. The total number of radiographic findings (the Pubic Symphysis Radiographic Severity Score) was not associated with groin pain. In the group of symptomatic male football players, the PSRS Score showed a weak negative correlation to HAGOS Pain and HAGOS Quality of life, while bone lucency and joint space narrowing were associated with lower pain intensity, and sclerosis was associated with higher disability.

### 4.1 Pubic‐Related Radiographic Findings

Radiographic findings of erosion, cysts, bone proliferation, and sclerosis are common degenerative radiographic findings related to joint diseases. When these findings are present at the pubic symphysis joint and surrounding bone in male athletes with adductor and pubic‐related groin pain, they have been interpreted as degenerative changes that may contribute to pain [2, 7, 8, 9, 10, 11, 12, 13, 14, 15, 16, 17]. The prevalence of each finding is rarely reported, but erosions, cysts, and sclerosis are often described as classical features of athletes with pubic and/or adductor‐related groin pain [7, 8, 9, 10, 11, 12, 13, 14, 15, 16, 17]. These athletes are also described with joint space changes (narrowing and widening), which likely result from erosions [9, 10, 13, 20], or proliferations [22]. Fragmentations at the pubic symphysis are less frequently reported, but it has been suggested that fragmentations may represent specific conditions such as apophysitis or bone maturation status [22, 34].

With the Aspetar protocol, we used a clear and reliable method for assessing each of these radiographic findings independently, and with a higher level of detail and methodological rigor than ever before reported by previous studies. Importantly, this study included both asymptomatic football players and non‐football athletes matched on age and physical activity level to the symptomatic athletes, which allows for determining whether pubic‐related radiographic findings may be attributed to groin pain or playing football [24]. Between football players and non‐football players, the absolute prevalence differences ranged between 8% and 50% and were statistically significant for 9 of the 15 main and subclassification findings. However, there were no statistically significant differences between football players with and without groin pain, despite rather clear differences for central/superior ELC (21%points), cysts (20%points), sclerosis (14% points), narrow pubic joint space (11% points) and fragmentations (9% points). These results suggest that many of the pubic‐related radiographic findings, from the Aspetar protocol, may be attributed to playing football rather than groin pain. This assumption is in line with at least five studies that have also suggested that pubic‐related radiographic findings are associated with sports activity rather than groin pain [23, 35, 36, 37, 38].

It is important to note that 13 of 39 (33%) symptomatic players were not classified with adductor‐ or pubic‐related groin pain. This raises a concern of false‐negative findings (type two error) if the radiographic findings truly are pathognomonic for adductor‐ and pubic‐related groin pain [2, 7, 8, 9, 10, 11, 12, 13, 14, 15, 16, 17]. To address this, we performed a post hoc analyses comparing the asymptomatic groups to the 26 symptomatic football players with adductor or pubic‐related groin pain. This analysis produced similar results (Appendix Tables A7 and A8). To improve the understanding of pathogenesis and mechanisms underlying especially adductor‐ and pubic‐related groin pain, future studies should explore other aspects of nociception and pain processing rather than focusing on specific radiological and surgical findings in the groin as the only factors contributing to long‐standing groin pain.

### 4.2 The Pubic Symphysis Radiographic Severity (PSRS) Score

The radiographic severity and progression of degenerative joint disease are often described with classification systems such as the Kellgren‐Lawrence grade [39]. Inspired by this, we developed four versions of a cumulative PSRS Score, as a pragmatic approach to quantify the extent and severity of pubic‐related radiographic findings. The results indicate that all versions of the PSRS score have limited value and clinical relevance. We found similar PSRS scores in symptomatic and asymptomatic players (see Figure 4), while in the symptomatic football players the PSRS Score correlated weakly to HAGOS Pain and QoL. However, the significant correlations diminished when the PSRS Score range increased from 0–5 to 0–18, and the *p* values were adjusted for false discovery rate. This suggests that the significant correlations should be interpreted with caution and confirmed by larger studies, as they are at risk of being random findings.

To improve the PSRS score and potentially enhance the relevance of a PSRS score in both research and clinical contexts, it may be beneficial to weight each finding independently or construct clusters of findings rather than applying an equal weight to all findings. This could be investigated further using methods such as principal component or factor analyses in data from larger samples. Another option could be to construct the PSRS score based on the findings with the largest differences between symptomatic and asymptomatic football players.

### 4.3 Influence of Individual Pubic‐Related Radiographic Findings on 5SST and HAGOS

When analyzing only the symptomatic players, sclerosis was associated with significantly worse scores on HAGOS Pain and ADL subscales. The football players with long‐standing groin pain and sclerosis scored approximately 20 points lower on HAGOS Pain and ADL compared to players without sclerosis. This difference exceeds the minimal important changes (MIC) thresholds for HAGOS Pain (9.7 points) and ADL subscales (11.8 points) [40], suggesting that sclerosis may be a clinically meaningful radiographic finding. Sclerosis may represent a specific mechanism or worsened state of groin pain, as it often presents at later stages of joint degeneration. These associations could therefore be influenced by age, duration of symptoms, and time spent participating in football.

The regression models showed that bony lucency and narrow joint space were associated with lower groin pain intensity as measured by the 5SST. This was an unexpected finding that contradicts the models for HAGOS subscales and the correlation between radiographic findings and HAGOS Pain. This discrepancy may stem from methodological limitations such as Type‐1 and Type‐2 errors, or the limited sample size. Alternatively, it can be speculated that bone lucency and narrow joint space reflect an earlier or later stage of groin pain, where the athletes and their pubic region are less sensitive to the mechanical forces during the 5SST.

### 4.4 Limitations

The classification of groin pain relied solely on the clinical pain provocation tests [32]. Even though the flexion adduction internal rotation (FADIR) test can effectively screen for hip‐joint related groin pain, relying only on clinical tests may result in underreporting of hip‐joint related groin pain in symptomatic athletes with a positive FADIR test and radiographic cam, pincer, borderline dysplasia, or dysplasia.

All radiographs were taken in a public hospital by multiple technicians. This reflects a real‐life clinic, although using a single technician may have reduced variation and error, as it is the technician's responsibility to secure satisfactory patient positioning. For the final dataset, we used consensus readings from two radiologists, with different levels of experience. This approach reduces the influence of individual skills and errors but limits generalizability, as single‐rater readings are common in clinical practice. However, consensus readings are still at risk of error and bias due to lack of variability, influence of the senior radiologist on the junior radiologist, “group thinking” and application of wrong assumptions [41, 42].

The interpretation of fragmentations, erosions, and cysts may have been influenced by the maturation status of the pubic symphysis [34], as this is not accounted for by the Aspetar protocol [22]. Van Ovost et al. have developed the maturation of the adolescent pubic symphysis (MAPS) classification method [34], which may enhance the radiographic assessment of the pubic symphysis joint when added to the Aspetar protocol.

The fixed sample size limited the ability to detect potentially clinically meaningful differences. For example, detecting a 21% difference in superior/central ELC between two groups would have required at least 78 participants per group. The large number of statistical analyses increased the risk of false positive findings (Type 1 errors). As this was highlighted during peer review, we added adjusted *p* values post hoc to allow readers to evaluate the risk of type 1 error.

## 5 Perspectives

While the Aspetar protocol is a reliable method for research and group‐level assessments, pubic‐related radiographic findings scored with the protocol and the cumulative PSRS Score have limited clinical relevance. The radiographic findings are common in male football players regardless of pain and do not add any valuable information about potential pathology in the pubic region.

At this point, radiographic examinations should be reserved for screening serious pathology, such as bony abnormalities, fractures, and sarcomas. Clinicians should be cautious if using pubic‐related radiographic findings in the diagnostic workup of groin pain.

Larger cross‐sectional and prospective cohort studies are still needed to confirm or refute these results, as findings from such studies may change the conclusion regarding the clinical relevance of the radiographic findings. This is particularly important for sclerosis, which showed a clinically relevant association with worse disability in the symptomatic football players with groin pain. The conclusion on clinical relevance may also be changed by future results about the progression of pubic‐related radiographic findings over time in both female and male athletes, the association with groin pain incidence, and the response to treatment.

## 6 Conclusion

In conclusion, pubic‐related radiographic findings are not associated with groin pain or disability. Pubic‐related radiographic findings are more common in male football players than in male non‐football athletes.

## Author Contributions

The first author is the lead and corresponding author. Contributions to the paper are described using the CRediT taxonomy by Brand A., Allen L., Altman M., Hlava M., and Scott J. (2015): conceptualization: M.F.N., P.H., and K.T.; methodology: M.F.N., P.H., S.B., T.T., L.I., M.B.N., and K.T.; formal analysis: M.F.N.; investigation: M.F.N., S.B., T.T., M.B.N., and K.T.; resources: P.H., S.B., T.T., M.B.N., and K.T.; data curation: M.F.N.; writing – original Draft: M.F.N. and K.T.; writing – review and editing manuscript: M.F.N., P.H., S.B., T.T., L.I., M.B.N., and K.T.; visualization: M.F.N.; supervision: PH, LI, and KT; project administration: M.F.N., P.H., and K.T.; funding acquisition: M.F.N., P.H., L.I., and K.T.

## Disclosure

Declaration of generative AI and AI‐assisted technologies in the writing process: During the preparation of this work the author used ChatGPT by OpenAI to trim the wordcount without loss of meaning as well as improve the grammar and readability. After using this tool/service, the author(s) reviewed and edited the content as needed and take(s) full responsibility for the content of the publication.

## Ethics Statement

This study has been approved by the Danish National Committee on Health Research Ethics (NVK 2117722) and the Capitol Region Data agency (P‐2021‐497). It includes data and radiographs collected in a larger epidemiological project on hip and/or groin pain (ethical approval reference: H‐2‐2010‐127, data approval reference: 2011‐41‐5964).

## Consent

Participants provided written informed consent when included in a previous larger epidemiological project on hip and/or groin pain (ethical approval reference: H‐2‐2010‐127, data approval reference: 2011‐41‐5964). The Danish National Committee on Health Research Ethics deemed that participants were not required to update their informed consent for this new project (NVK 2117722).

## Conflicts of Interest Statement

All authors declare they have no conflicts of interest. However, two authors (KT, PH) have conceived and published standardized clinical examinations of patients with long‐standing hip and/or groin pain, the Copenhagen Hip and Groin Outcome Score (HAGOS) and the Copenhagen 5‐s‐squeeze test. One author (PH) has contributed to the conception and publication of the Aspetar pubic symphysis radiographic scoring protocol. These authors are, therefore, subject to confirmation bias and self‐citation incentives.

## Supporting information


**Figure A1.** Scatterplot of 5‐Second‐Squeeze Test and PSRS Score 1 (0–5). Red line is a LOESS curve.
**Figure A2**. Scatterplot of 5‐Second‐Squeeze Test and PSRS Score 2 (0–8). Red line is a LOESS curve.
**Figure A3**. Scatterplot of 5‐Second‐Squeeze Test and PSRS Score 3 (0–10). Red line is a LOESS curve.
**Figure A4**. Scatterplot of 5‐Second‐Squeeze Test and PSRS Score 4 (0–18). Red line is a LOESS curve.
**Figure A5**. Scatterplot of HAGOS Pain and PSRS Score 1 (0–5). Red line is a LOESS curve.
**Figure A6**. Scatterplot of HAGOS Pain and PSRS Score 2 (0–8). Red line is a LOESS curve.
**Figure A7**. Scatterplot of HAGOS Pain and PSRS Score 3 (0–10). Red line is a LOESS curve.
**Figure A8**. Scatterplot of HAGOS Pain and PSRS Score 4 (0–18). Red line is a LOESS curve.
**Figure A9**. Scatterplot of HAGOS ADL and PSRS Score 1 (0–5). Red line is a LOESS curve.
**Figure A10**. Scatterplot of HAGOS ADL and PSRS Score 2 (0–8). Red line is a LOESS curve.
**Figure A11**. Scatterplot of HAGOS ADL and PSRS Score 3 (0–10). Red line is a LOESS curve.
**Figure A12**. Scatterplot of HAGOS ADL and PSRS Score 4 (0–18). Red line is a LOESS curve.
**Figure A13**. Scatterplots of HAGOS Quality of Life and PSRS Score 1 (0–5). Red line is a LOESS curve.
**Figure A14**. Scatterplots of HAGOS Quality of Life and PSRS Score 2 (0–8). Red line is a LOESS curve.
**Figure A15**. Scatterplots of HAGOS Quality of Life and PSRS Score 3 (0–10). Red line is a LOESS curve.
**Figure A16**. Scatterplots of HAGOS Quality of Life and PSRS Score 4 (0–18). Red line is a LOESS curve.

Data S1.

## Data Availability

The data that support the findings of this study are available on request from the corresponding author. The data are not publicly available due to privacy or ethical restrictions.

## References

[1] K. Thorborg , M. P. Reiman , A. Weir , et al., “Clinical Examination, Diagnostic Imaging, and Testing of Athletes With Groin Pain: An Evidence‐Based Approach to Effective Management,” Journal of Orthopaedic and Sports Physical Therapy 48, no. 4 (2018): 239–249, 10.2519/jospt.2018.7850. 29510653

[2] G. M. Verrall , L. Henry , N. L. Fazzalari , J. P. Slavotinek , and R. D. Oakeshott , “Bone Biopsy of the Parasymphyseal Pubic Bone Region in Athletes With Chronic Groin Injury Demonstrates New Woven Bone Formation Consistent With a Diagnosis of Pubic Bone Stress Injury,” American Journal of Sports Medicine 36, no. 12 (2008): 2425–2431, 10.1177/0363546508324690. 18927251

[3] A. Weir , P. Brukner , E. Delahunt , et al., “Doha Agreement Meeting on Terminology and Definitions in Groin Pain in Athletes,” British Journal of Sports Medicine 49, no. 12 (2015): 768–774, 10.1136/bjsports-2015-094869. 26031643 PMC4484366

[4] I. Becker , S. J. Woodley , and M. D. Stringer , “The Adult Human Pubic Symphysis: A Systematic Review,” Journal of Anatomy 217, no. 5 (2010): 475–487, 10.1111/j.1469-7580.2010.01300.x. 20840351 PMC3035856

[5] M. Kormano , “Radiographic Appearance of the Pubic Symphysis in Old Age and in Rheumatoid Arthritis,” Acta Rheumatologica Scandinavica 17, no. 1–4 (1971): 286–294, 10.3109/rhe1.1971.17.issue-1-4.39. 5156885

[6] V. A. Vix and C. Y. RYU , “The Adult Symphysis Pubis: Normal and Abnormal,” American Journal of Roentgenology 112, no. 3 (1971): 517–525, 10.2214/ajr.112.3.517. 5570362

[7] M. L. Amer , K. Omar , S. Malde , R. Nair , R. Thurairaja , and M. S. Khan , “The Challenges in Diagnosis and Management of Osteitis Pubis: An Algorithm Based on Current Evidence,” BJUI Compass 3, no. 4 (2022): 267–276, 10.1002/bco2.127. 35783593 PMC9231671

[8] J. J. Le Jeune , P. Rochcongar , F. Vazelle , A. M. Bernard , J. Y. Herry , and A. Ramée , “Pubic Pain Syndrome in Sportsmen: Comparison of Radiographic and Scintigraphic Findings,” European Journal of Nuclear Medicine 9, no. 6 (1984): 250–253, 10.1007/BF00803244. 6745295

[9] A. M. Shaker , M. A. Shaheen , and P. J. O'neel , “Traumatic Aseptic Osteitis Pubis,” Annals of Saudi Medicine 11, no. 2 (1991): 205–208, 10.5144/0256-4947.1991.205. 17588083

[10] M. A. Holt , J. S. Keene , B. K. Graf , and D. C. Helwig , “Treatment of Osteitis Pubis in Athletes: Results of Corticosteroid Injections,” American Journal of Sports Medicine 23, no. 5 (1995): 601–606, 10.1177/036354659502300515. 8526278

[11] P. R. Williams , D. P. Thomas , and E. M. Downes , “Osteitis Pubis and Instability of the Pubic Symphysis: When Nonoperative Measures Fail,” American Journal of Sports Medicine 28, no. 3 (2000): 350–355, 10.1177/03635465000280031101. 10843126

[12] C. Rodriguez , A. Miguel , H. Lima , and K. Heinrichs , “Osteitis Pubis Syndrome in the Professional Soccer Athlete: A Case Report,” Journal of Athletic Training 36, no. 4 (2001): 437. 12937486 PMC155442

[13] M. J. O'Connell , T. Powell , N. M. McCaffrey , D. O'Connell , and S. J. Eustace , “Symphyseal Cleft Injection in the Diagnosis and Treatment of Osteitis Pubis in Athletes,” American Journal of Roentgenology 179, no. 4 (2002): 955–959, 10.2214/ajr.179.4.1790955. 12239045

[14] K. S. Hechtman , J. E. Zvijac , C. A. Popkin , G. A. Zych , and A. van Botto‐ Bemden , “A Minimally Disruptive Surgical Technique for the Treatment of Osteitis Pubis in Athletes,” Sports Health 2, no. 3 (2010): 211–215, 10.1177/1941738110366203. 23015940 PMC3445106

[15] H. D. E. Atkinson , P. Johal , M. S. Falworth , V. S. Ranawat , B. Dala‐Ali , and D. K. Martin , “Adductor Tenotomy: Its Role in the Management of Sports‐Related Chronic Groin Pain,” Archives of Orthopaedic and Trauma Surgery 130, no. 8 (2010): 965–970, 10.1007/s00402-009-1032-4. 20033698

[16] S. J. Hopp , U. Culemann , J. Kelm , T. Pohlemann , and A. Pizanis , “Osteitis Pubis and Adductor Tendinopathy in Athletes: A Novel Arthroscopic Pubic Symphysis Curettage and Adductor Reattachment,” Archives of Orthopaedic and Trauma Surgery 133, no. 7 (2013): 1003–1009, 10.1007/s00402-013-1777-7. 23689650

[17] S. Branci , K. Thorborg , M. B. Nielsen , and P. Hölmich , “Radiological Findings in Symphyseal and Adductor‐Related Groin Pain in Athletes: A Critical Review of the Literature,” British Journal of Sports Medicine 47, no. 10 (2013): 611–619, 10.1136/bjsports-2012-091905. 23403531

[18] N. H. Harris and R. O. Murray , “Lesions of the Symphysis in Athletes,” British Medical Journal 4, no. 5938 (1974): 211–214. 4422968 10.1136/bmj.4.5938.211 PMC1612396

[19] D. Brennan , M. J. O'Connell , M. Ryan , et al., “Secondary Cleft Sign as a Marker of Injury in Athletes With Groin Pain: MR Image Appearance and Interpretation,” Radiology 235, no. 1 (2005): 162–167, 10.1148/radiol.2351040045. 15731372

[20] J. Besjakov , C. von Scheele , O. Ekberg , C. F. Gentz , and N. E. Westlin , “Grading Scale of Radiographic Findings in the Pubic Bone and Symphysis in Athletes,” Acta Radiologica Stockholm Sweden 1987 44, no. 1 (2003): 79–83. 12631004

[21] N. M. Major and C. A. Helms , “Pelvic Stress Injuries: The Relationship Between Osteitis Pubis (Symphysis Pubis Stress Injury) and Sacroiliac Abnormalities in Athletes,” Skeletal Radiology 26, no. 12 (1997): 711–717, 10.1007/s002560050316. 9453104

[22] A. Serner , J. Arnaiz , A. Mosler , et al., “Classifying Radiographic Changes of the Pubic Symphysis in Male Athletes: Development and Reproducibility of a New Scoring Protocol,” European Journal of Radiology 134 (2021): 109452, 10.1016/j.ejrad.2020.109452. 33310551

[23] M. A. Martens , L. Hansen , and J. C. Mulier , “Adductor Tendinitis and Musculus Rectus Abdominis Tendopathy,” American Journal of Sports Medicine 15, no. 4 (1987): 353–356, 10.1177/036354658701500410. 2959165

[24] S. Branci , K. Thorborg , B. H. Bech , M. Boesen , M. B. Nielsen , and P. Hölmich , “MRI Findings in Soccer Players With Long‐Standing Adductor‐Related Groin Pain and Asymptomatic Controls,” British Journal of Sports Medicine 49, no. 10 (2015): 681–691, 10.1136/bjsports-2014-093710. 25512059

[25] K. Thorborg , M. S. Rathleff , P. Petersen , S. Branci , and P. Hölmich , “Prevalence and Severity of Hip and Groin Pain in Sub‐Elite Male Football: A Cross‐Sectional Cohort Study of 695 Players,” Scandinavian Journal of Medicine & Science in Sports 27, no. 1 (2017): 107–114, 10.1111/sms.12623. 26643978

[26] S. Branci , K. Thorborg , B. H. Bech , et al., “The Copenhagen Standardised MRI Protocol to Assess the Pubic Symphysis and Adductor Regions of Athletes: Outline and Intratester and Intertester Reliability,” British Journal of Sports Medicine 49, no. 10 (2015): 692–699, 10.1136/bjsports-2014-094239. 25488954

[27] M. F. Nielsen , P. Hölmich , S. Branci , et al., “Intra‐ and Inter‐Rater Reliability of Hip‐ and Pubic‐Related Radiographic Parameters in 86 Male Athletes,” Scandinavian Sports Medicine Congress; Presented as Abstract 72 at: Poster Walk ‐ Muscle and Exercise: Innovative Approaches (2025).

[28] K. Thorborg , S. Branci , M. P. Nielsen , M. T. Langelund , and P. Hölmich , “Copenhagen Five‐Second Squeeze: A Valid Indicator of Sports‐Related Hip and Groin Function,” British Journal of Sports Medicine 51, no. 7 (2017): 594–599, 10.1136/bjsports-2016-096675. 27935487

[29] K. Thorborg , P. Hölmich , R. Christensen , J. Petersen , and E. M. Roos , “The Copenhagen Hip and Groin Outcome Score (HAGOS): Development and Validation According to the COSMIN Checklist,” British Journal of Sports Medicine 45, no. 6 (2011): 478–491, 10.1136/bjsm.2010.080937. 21478502

[30] K. B. Christensen , M. B. Clausen , E. King , et al., “Validation of the Copenhagen Hip and Groin Outcome Score (HAGOS) Using Modern Test Theory Across Different Cultures and Languages: A Cross‐Sectional Study of 452 Male Athletes With Groin Pain,” British Journal of Sports Medicine 56, no. 6 (2022): 104412, 10.1136/bjsports-2021-104412. 34815222

[31] P. Hölmich , “Clinical Examination of Athletes With Groin Pain: An Intraobserver and Interobserver Reliability Study,” British Journal of Sports Medicine 38, no. 4 (2004): 446–451, 10.1136/bjsm.2003.004754. 15273182 PMC1724872

[32] W. M. P. Heijboer , A. Weir , Z. Vuckovic , et al., “Inter‐Examiner Reliability of the Doha Agreement Meeting Classification System of Groin Pain in Male Athletes,” Scandinavian Journal of Medicine & Science in Sports 33, no. 2 (2023): 189–196, 10.1111/sms.14248. 36259124 PMC10092143

[33] Y. Benjamini and Y. Hochberg , “Controlling the False Discovery Rate: A Practical and Powerful Approach to Multiple Testing,” Journal of the Royal Statistical Society: Series B: Methodological 57, no. 1 (1995): 289–300, 10.1111/j.2517-6161.1995.tb02031.x.

[34] A. van Ovost , D. F. Hanff , A. Serner , P. van Klij , R. Agricola , and A. Weir , “Radiographic Assessment of the Pubic Symphysis in Elite Male Adolescent Football Players: Development and Reliability of the Maturing Adolescent Pubic Symphysis (MAPS) Classification,” European Journal of Radiology 167 (2023): 111068, 10.1016/j.ejrad.2023.111068. 37666074

[35] D. C. Taylor , W. C. Meyers , J. A. Moylan , J. Lohnes , F. H. Bassett , and W. E. Garrett , “Abdominal Musculature Abnormalities as a Cause of Groin Pain in Athletes: Inguinal Hernias and Pubalgia,” American Journal of Sports Medicine 19, no. 3 (1991): 239–242, 10.1177/036354659101900306. 1831010

[36] A. L. Polglase , G. M. Frydman , and K. C. Farmer , “Inguinal Surgery for Debilitating Chronic Groin Pain in Athletes,” Medical Journal of Australia 155, no. 10 (1991): 674–677. 1943896

[37] R. N. van Veen , P. de Baat , M. P. Heijboer , et al., “Successful Endoscopic Treatment of Chronic Groin Pain in Athletes,” Surgical Endoscopy 21, no. 2 (2007): 189–193, 10.1007/s00464-005-0781-6. 17122983

[38] P. Ziprin , S. G. Prabhudesai , S. Abrahams , and S. J. Chadwick , “Transabdominal Preperitoneal Laparoscopic Approach for the Treatment of Sportsman's Hernia,” Journal of Laparoendoscopic & Advanced Surgical Techniques 18, no. 5 (2008): 669–672, 10.1089/lap.2007.0130. 18699749

[39] J. N. Katz , K. R. Arant , and R. F. Loeser , “Diagnosis and Treatment of Hip and Knee Osteoarthritis: A Review,” Journal of the American Medical Association 325, no. 6 (2021): 568–578, 10.1001/jama.2020.22171. 33560326 PMC8225295

[40] R. Thomeé , P. Jónasson , K. Thorborg , et al., “Cross‐Cultural Adaptation to Swedish and Validation of the Copenhagen Hip and Groin Outcome Score (HAGOS) for Pain, Symptoms and Physical Function in Patients With Hip and Groin Disability due to Femoro‐Acetabular Impingement,” Knee Surgery, Sports Traumatology, Arthroscopy 22, no. 4 (2014): 835–842, 10.1007/s00167-013-2721-7. 24146052

[41] A. A. Bankier , D. Levine , E. F. Halpern , and H. Y. Kressel , “Consensus Interpretation in Imaging Research: Is There a Better Way?,” Radiology 257, no. 1 (2010): 14–17, 10.1148/radiol.10100252. 20851935

[42] N. A. Obuchowski and R. C. Zepp , “Simple Steps for Improving Multiple‐Reader Studies in Radiology,” American Journal of Roentgenology 166, no. 3 (1996): 517–521, 10.2214/ajr.166.3.8623619. 8623619

