# Supplementary material for: Pubic‐Related Radiographic Findings in Male Football Players With Long‐Standing Groin Pain, and Asymptomatic Controls — Are They Clinically Relevant?
Source: Scand J Med Sci Sports. 2025 May 13;35(5):e70068. doi: 10.1111/sms.70068 (PMC12070245; doi:10.1111/sms.70068)
Supplement: Supplementary file 1 — Figure A1. Scatterplot of 5‐Second‐Squeeze Test and PSRS Score 1 (0–5). Red line is a LOESS curve. Figure A2. Scatterplot of 5‐Second‐Squeeze Test and PSRS Score 2 (0–8). Red line is a LOESS curve. Figure A3. Scatterplot of 5‐Second‐Squeeze Test and PSRS Score 3 (0–10). Red line is a LOESS curve. Figure A4. Scatterplot of 5‐Second‐Squeeze Test and PSRS Score 4 (0–18). Red line is a LOESS curve. Figure A5. Scatterplot of HAGOS Pain and PSRS Score 1 (0–5). Red line is a LOESS curve. Figure A6. Scatterplot of HAGOS Pain and PSRS Score 2 (0–8). Red line is a LOESS curve. Figure A7. Scatterplot of HAGOS Pain and PSRS Score 3 (0–10). Red line is a LOESS curve. Figure A8. Scatterplot of HAGOS Pain and PSRS Score 4 (0–18). Red line is a LOESS curve. Figure A9. Scatterplot of HAGOS ADL and PSRS Score 1 (0–5). Red line is a LOESS curve. Figure A10. Scatterplot of HAGOS ADL and PSRS Score 2 (0–8). Red line is a LOESS curve. Figure A11. Scatterplot of HAGOS ADL and PSRS Score 3 (0–10). Red line is a LOESS curve. Figure A12. Scatterplot of HAGOS ADL and PSRS Score 4 (0–18). Red line is a LOESS curve. Figure A13. Scatterplots of HAGOS Quality of Life and PSRS Score 1 (0–5). Red line is a LOESS curve. Figure A14. Scatterplots of HAGOS Quality of Life and PSRS Score 2 (0–8). Red line is a LOESS curve. Figure A15. Scatterplots of HAGOS Quality of Life and PSRS Score 3 (0–10). Red line is a LOESS curve. Figure A16. Scatterplots of HAGOS Quality of Life and PSRS Score 4 (0–18). Red line is a LOESS curve. [file SMS-35-e70068-s002.docx]

**Appendix file**

**Content**

1. Descriptives data of HAGOS original version.
2. Pairwise comparison of pubic-related findings – side-specific level.
3. Pubic Symphysis Radiographic Severity Scores compared between groups.
4. Correlation coefficients for associations between Pubic Symphysis Radiographic Severity (PSRS) Scores and 5SST and HAGOS Subscales in the symptomatic football players (n=39)
5. Examples of scatterplots for associations between Pubic Symphysis Radiographic Severity (PSRS) Scores and 5SST and HAGOS Subscales in the symptomatic football players (n=39)
6. Linear regression models for associations between Pubic Symphysis Radiographic Severity (PSRS) Scores and 5SST and HAGOS Subscales in the symptomatic football players (n=39)
7. Post-hoc analyses including only symptomatic football players with adductor- or pubic-related groin pain
8. Post-hoc adjusted p-values for prevalence estimates.
9. **Descriptives**

| **TABLE A1 – Descriptive variables including original HAGOS, clinical entities and hip-related radiographic findings** | | | |
| --- | --- | --- | --- |
|  | **Symptomatic**  **football players**  **(n = 39)** | **Asymptomatic**  **football players**  **(n = 18)** | **Asymptomatic**  **active athletes**  **(n = 20)** |
| **HAGOS** |  |  |  |
| *Pain, median (IQR)* | 75 (62-85) | 100 (95-100) | 100 (99-100) |
| *Symptoms, median (IQR)* | 61 (50-71) | 93 (82-96) | 96 (89-100) |
| *ADL, median (IQR)* | 80 (65-90) | 100 (100-100) | 100 (100-100) |
| *Sport, median (IQR)* | 50 (34-62) | 100 (94-100) | 100 (100-100) |
| *PA, median (IQR)* | 37 (19-62) | 100 (100-100) | 100 (100-100) |
| *QoL, median (IQR)* | 40 (27-60) | 100 (90-100) | 100 (100-100) |
| **Clinical entities of groin pain** | **Individual level** |  |  |
| Adductor-related groin pain, n (%) | 26 (67%) | - | - |
| Iliopsoas-related groin pain, n (%) | 31 (79%) | - | - |
| Inguinal-related groin pain, n (%) | 24 (61%) | - | - |
| Pubic-related groin pain, n (%) | 5 (13%) | - | - |
| Total entities, median (IQR) | 2 (2-3) | - | - |
| **Hip-related Radiographic Findings** | **Individual level** | **Individual level** | **Individual level** |
| Alpha Angle, mean (SD) | 54 (10) | 52 (12) | 48 (10) |
| *Cam morphology*, n (%) | 12 (31%) | 6 (33%) | 4 (20%) |
| Lateral Center Edge Angle, mean (SD) | 30 (5) | 28 (8) | 31 (5) |
| Acetabular Index Angle, mean (SD) | 6 (4) | 9 (4) | 6 (4) |
| *Pincer Morphology, n (%)* | 3 (12%) | 1 (10%) | 1 (7%) |
| *Borderline Hip Dysplasia, n (%)* | 7 (29%) | 4 (40%) | 2 (13%) |
| *Hip Dysplasia, n (%)* | 3 (12%) | 4 (40%) | 1 (7%) |
| Hip joint space width, mm, mean (SD) |  |  |  |
| Cross-Over sign, n (%) | 16 (67%) | 6 (60%) | 8 (53%) |
| Ischial Spine Sign, n (%) | 19 (79%) | 6 (60%) | 11 (73%) |
| Posterior Wall Sign, n (%) | 19 (79%) | 7 (70%) | 11 (73%) |

1. **Pairwise comparison of pubic-related findings – side-specific level**

| **Table A2 Prevalence of pubic-related findings on side-level and pairwise comparison between groups** | | | | | | |
| --- | --- | --- | --- | --- | --- | --- |
|  | **Symptomatic**  **football players,**  *painful sides* | | **Asymptomatic**  **football players** | | **Asymptomatic**  **non-football athletes**  *n = 20* | |
| **Pubic-related radiographic finding, n (%)** | *Right,*  *n =32* | *Left,*  *n =23* | *Right,*  *n = 18* | *Left,*  *n = 18* | *Right,*  *n = 20* | *Left,*  *n = 20* |
| **Bone Lucency** | 26 (81%) | 18 (78%) | 11 (61%) | 12 (67%) | 8 (40%)** | 7 (35%)** |
| *Erosion-Like Configuration* | 26 (81%) | 18 (78%) | 11 (61%) | 12 (67%) | 8 (40%)** | 7 (35%)** |
| Superior/Central ELC | 22 (69%) | 16 (70%) | 8 (44%) | 10 (56%) | 6 (30%)* | 7 (35%)* |
| Inferior ELC | 22 (69%) | 16 (70%) | 10 (56%) | 11 (61%) | 5 (25%)** | 6 (30%)* |
| *Cysts* | 5 (16%) | 6 (26%) | 2 (11%) | 1 (6%) | 0 (0%) | 2 (10%) |
| **Proliferation** | 17 (53%) | 14 (61%) | 11 (61%) | 10 (56%) | 4 (20%)* | 4 (20%)* |
| *Superior Proliferation* | 15 (47%) | 11 (48%) | 10 (56%) | 7 (39%) | 3 (15%)* | 3 (15%)* |
| *Central Proliferation* | 8 (25%) | 7 (30%) | 6 (33%) | 5 (28%) | 1 (5%) | 1 (5%) |
| *Inferior Proliferation* | 5 (16%) | 4 (17%) | 5 (28%) | 4 (22%) | 1 (5%) | 0 (0%) |
| **Fragmentations** | 2 (6%) | 4 (17%) | 0 (0%) | 1 (6%) | 0 (0%) | 0 (0%) |
| *Central Fragmentation* | 1 (3%) | 2 (9%) | 0 (0%) | 1 (6%) | 0 (0%) | 0 (0%) |
| *Inferior Fragmentation* | 2 (6%) | 2 (9%) | 0 (0%) | 0 (0%) | 0 (0%) | 0 (0%) |
| **Sclerosis** | 17 (53%) | 13 (57%) | 8 (44%) | 9 (50%) | 2 (10%)** | 3 (15%)* |
| **Statistically significantly different compared to symptomatic football players at a p <0.05 level*  ***Statistically significantly different compared to symptomatic football players at a p <0.005 level* | | | | | | |

1. **Pubic Symphysis Radiographic Severity Scores between groups**

| **Table A3 Between group comparisons of Pubic Symphysis Radiographic Severity (PSRS) Scores** | | | | | | | | | | | | | | | | | | |
| --- | --- | --- | --- | --- | --- | --- | --- | --- | --- | --- | --- | --- | --- | --- | --- | --- | --- | --- |
|  | **Symptomatic**  **football**  **players**  **(n = 39)** | | **Asymptomatic**  **football**  **players**  **(n=17)** | | **Asymptomatic**  **non-football**  **athletes**  **(n=20)** | | **Symptomatic football**  **vs**  **Asymptomatic football** | | | | **Symptomatic football**  **Vs**  **Asymptomatic non-football** | | | | **Asymptomatic football**  **Vs**  **Asymptomatic non-football** | | | |
| **PSRS Scores** | ***Mean*** | ***(SD)*** | ***Mean*** | ***(SD)*** | ***Mean*** | ***(SD)*** | **Diff** | ***(95% CI)*** | ***p-value*** | **Adj. P** | **Diff** | ***(95% CI)*** | ***p-value*** | **Adj. P** | **Diff** | ***(95% CI)*** | ***p-value*** | **Adj. P** |
| PSRS Score 1 (0-5) | 2.6 | (1.4) | 2.2 | (1.4) | 1.0 | (1.2) | 0.4 | (–0.5 to 1.4) | 0.27 | 0.27 | 1.6 | (0.7 to 2.5) | **< 0.00** | **< 0.00** | 1.2 | (0.1 to 2.2) | **0.009** | **0.01** |
| PSRS Score 2 (0-8) | 4.1 | (2.2) | 3.4 | (2.2) | 1.4 | (1.7) | 0.7 | (–0.9 to 2.2) | 0.30 | 0.30 | 2.7 | (1.4 to 4.0) | **< 0.00** | **< 0.00** | 2.0 | (0.5 to 3.6) | **0.003** | **0.004** |
| PSRS Score 3 (0-10) | 4.4 | (2.5) | 3.6 | (2.5) | 1.5 | (1.8) | 0.8 | (–0.9 to 2.6) | 0.26 | 0.26 | 2.9 | (1.5 to 4.3) | **< 0.00** | **< 0.00** | 2.1 | (0.3 to 3.8) | **0.007** | **0.01** |
| PSRS Score 4 (0-18) | 6.4 | (3.6) | 5.4 | (3.9) | 2.0 | (2.6) | –1.0 | (–1.6 to 3.6) | 0.37 | 0.37 | 4.4 | (2.4 to 6.4) | **< 0.00** | **< 0.00** | 3.4 | (0.7 to 6.1) | **0.003** | **0.005** |
| *PSRS = Pubic Symphysis Radiographic Severity, SD = standard deviation, CI = confidence interval, Adj. p = Benjamini-Hochberg adjusted p-values* | | | | | | | | | | | | | | | | | | |

1. **Correlation coefficients**

| **Table A4 Correlation coefficients for associations between Pubic Symphysis Radiographic Severity (PSRS) Scores and 5SST and HAGOS Subscales**  **in the symptomatic football players (n=39)** | | | | | | | | | | | | | | | | |
| --- | --- | --- | --- | --- | --- | --- | --- | --- | --- | --- | --- | --- | --- | --- | --- | --- |
|  | **PSRS Score 1 (0-5)** | | | | **PSRS Score 2 (0-8)** | | | | **PSRS Score 3 (0-10)** | | | | **PSRS Score 4 (0-18)** | | | |
|  | ***ρ*** | ***(95% CI)*** | ***p-value*** | ***Ajd. p*** | ***ρ*** | ***(95% CI)*** | ***p-value*** | ***Ajd. p*** | ***ρ*** | ***(95% CI)*** | ***p-value*** | ***Ajd. p*** | ***ρ*** | ***(95% CI)*** | ***p-value*** | ***Ajd. p*** |
| **5-Second Squeeze Test** | –0.10 | (–0.41 to 0.23) | 0.53 | 0.61 | 0.05 | (–0.28 to 0.37) | 0.75 | 0.85 | –0.16 | (–0.46 to 0.17) | 0.33 | 0.78 | –0.12 | (–0.43 to 0.21) | 0.46 | 0.80 |
| **HAGOS,** Revised version |  | | | | | | | | | | | | | | | |
| *Pain* | **–0.40** | **(–0.64 to –0.09)** | **0.01** | 0.08 | **–0.39** | **(–0.63 to –0.07)** | **0.01** | 0.10 | –0.24 | (–0.53 to 0.09) | 0.14 | 0.48 | –0.14 | (–0.45 to 0.19) | 0.39 | 0.80 |
| *Symptoms* | –0.15 | (–0.45 to 0.18) | 0.36 | 0.50 | –0.05 | (–0.36 to 0.28) | 0.78 | 0.85 | 0.00 | (–0.33 to 0.32) | 0.98 | 0.98 | 0.09 | (–0.24 to 0.40) | 0.57 | 0.80 |
| *ADL* | –0.21 | (–0.50 to 0.12) | 0.20 | 0.47 | –0.24 | (–0.52 to 0.09) | 0.14 | 0.32 | –0.05 | (–0.37 to 0.28) | 0.75 | 0.98 | –0.05 | (–0.36 to 0.28) | 0.78 | 0.91 |
| *SPORT* | –0.17 | (–0.47 to 0.16) | 0.29 | 0.50 | –0.17 | (–0.47 to 0.16) | 0.29 | 0.51 | –0.05 | (–0.37 to 0.28) | 0.75 | 0.98 | –0.01 | (–0.33 to 0.32) | 0.95 | 0.95 |
| *PA* | –0.01 | (–0.33 to 0.31) | 0.95 | 0.95 | 0.03 | (–0.30 to 0.35) | 0.85 | 0.85 | 0.03 | (–0.30 to 0.35) | 0.87 | 0.98 | 0.12 | (–0.21 to 0.43) | 0.47 | 0.80 |
| *QoL* | **–0.34** | **(–0.60 to –0.01)** | **0.04** | 0.13 | –0.28 | (–0.56 to 0.05) | 0.08 | 0.28 | –0.28 | (–0.55 to 0.05) | 0.09 | 0.48 | –0.19 | (–0.49 to 0.14) | 0.24 | 0.80 |
| *ρ = Spearmans Correlations Coefficients, 95% CI = 95% Confidence Interval, adj. p = Benjamini-Hocberg adjusted p-value* | | | | | | | | | | | | | | | | |

1. **Examples of scatterplots for associations between Pubic Symphysis Radiographic Severity (PSRS) Scores and 5SST and HAGOS Subscales in the symptomatic football players (n=39)**


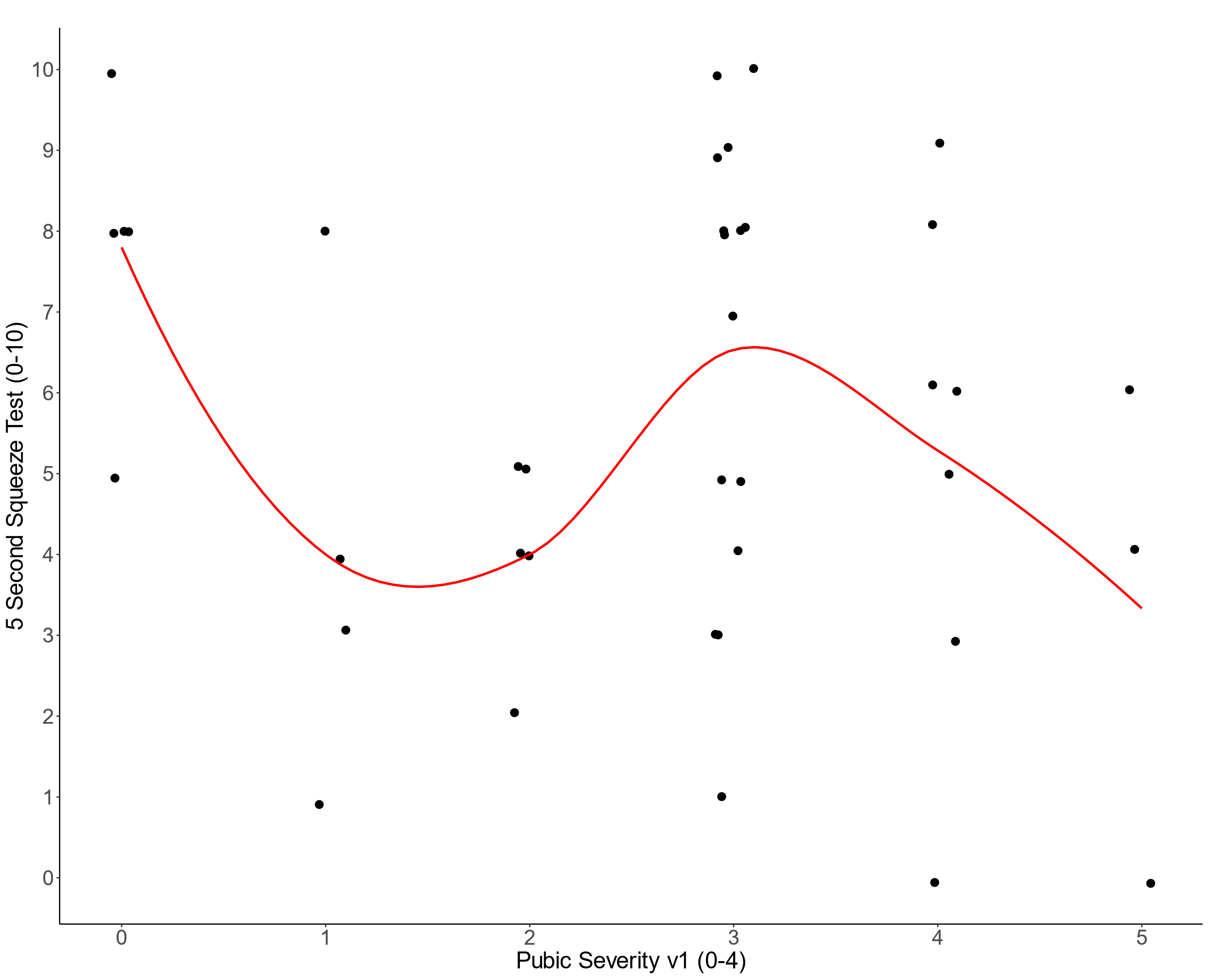


**Figure A1 Scatterplot of 5-Second-Squeeze Test and PSRS Score 1 (0-5).** Red line is a LOESS curve.


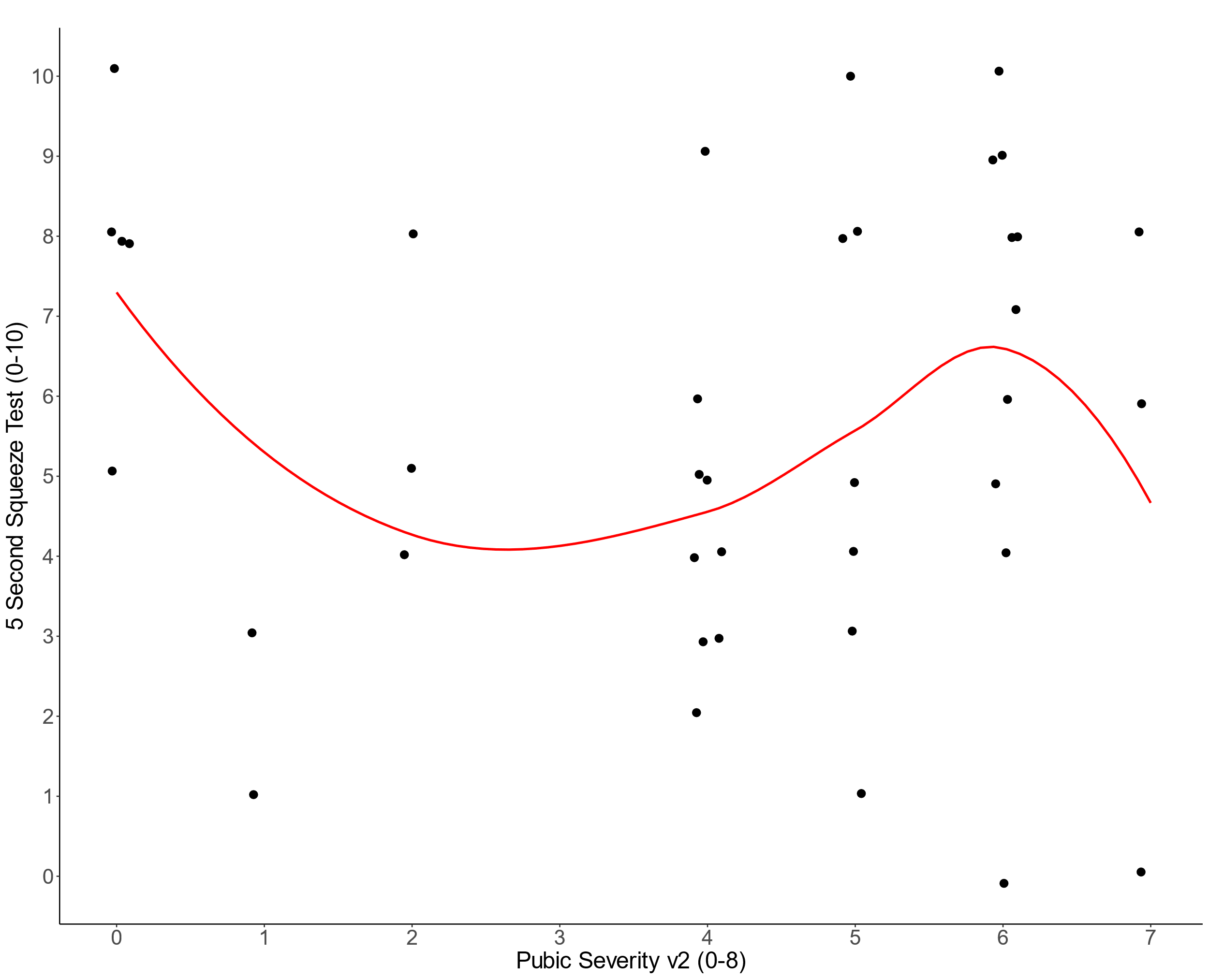


**Figure A2 Scatterplot of 5-Second-Squeeze Test and PSRS Score 2 (0-8).** Red line is a LOESS curve.


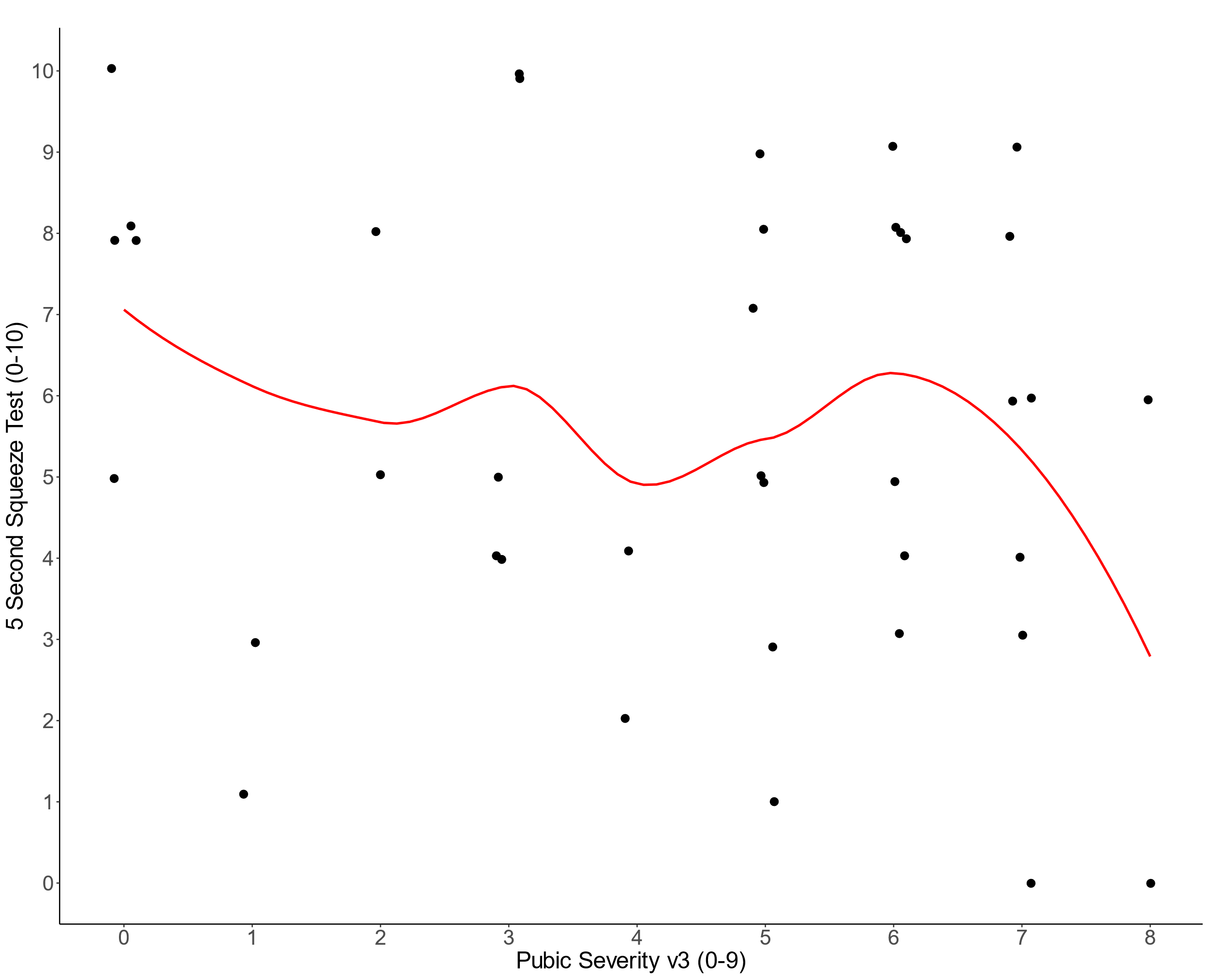


**Figure A3 Scatterplot of 5-Second-Squeeze Test and PSRS Score 3 (0-10).** Red line is a LOESS curve.


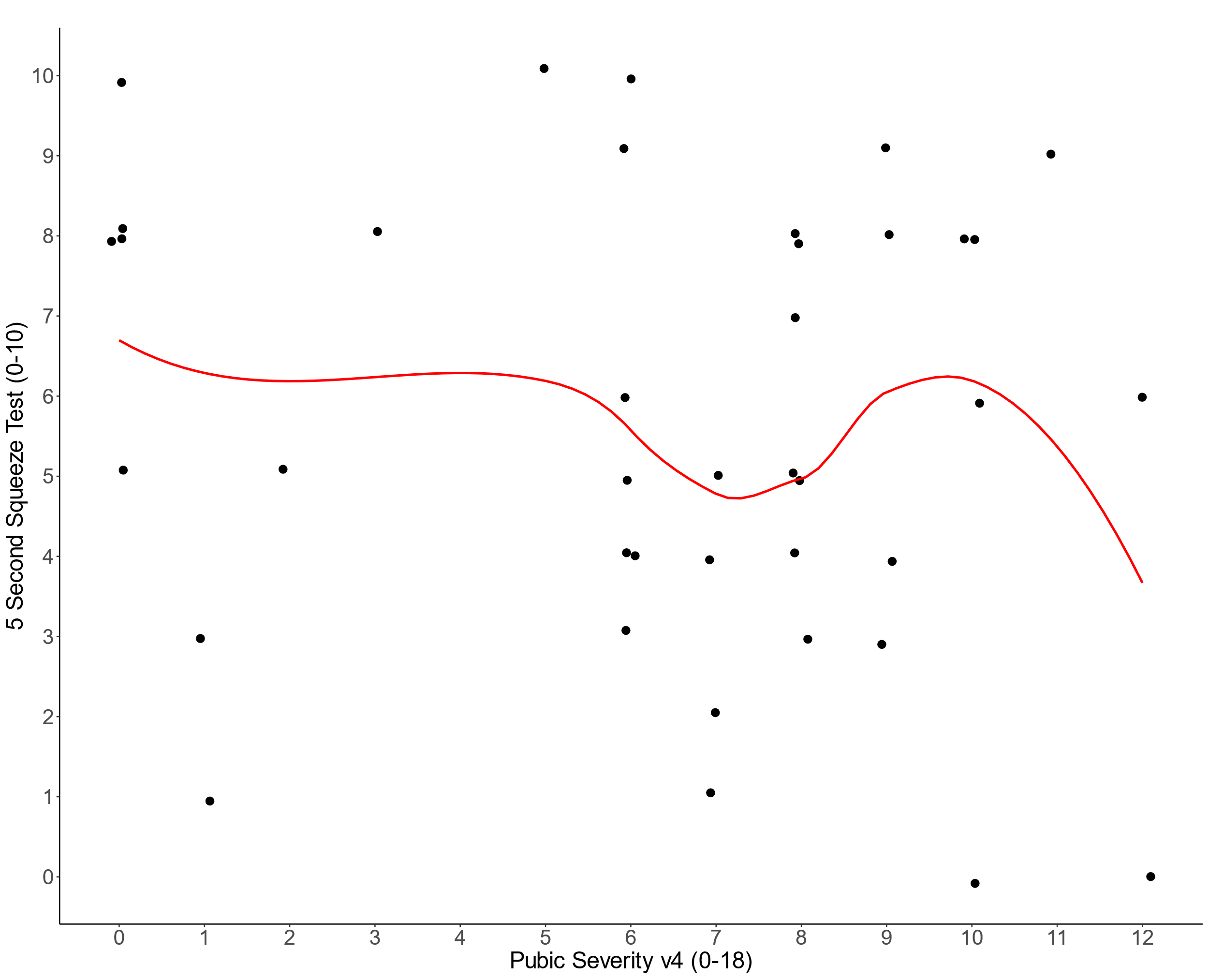


**Figure A4 Scatterplot of 5-Second-Squeeze Test and PSRS Score 4 (0-18).** Red line is a LOESS curve.


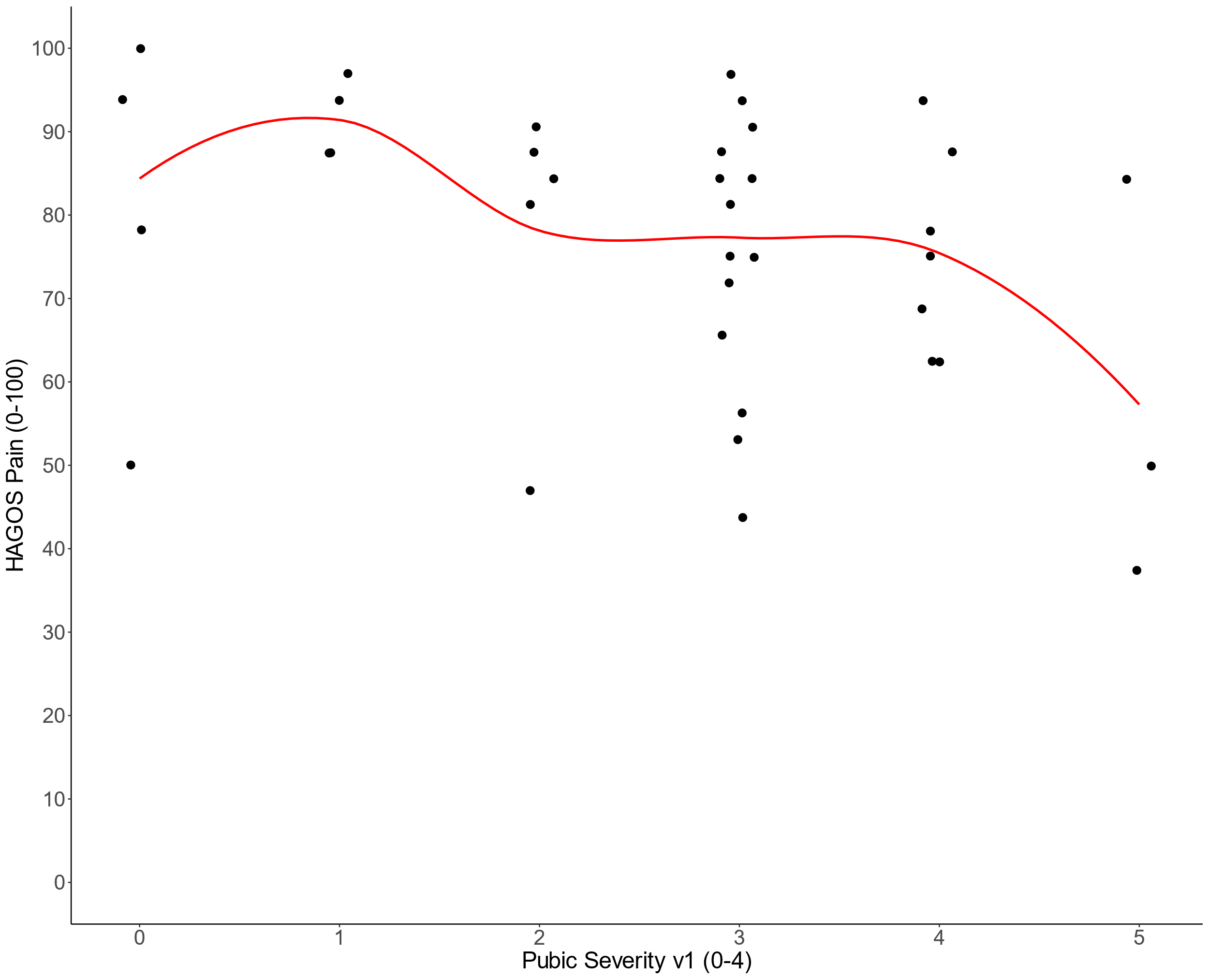


**Figure A5 Scatterplot of HAGOS Pain and PSRS Score 1 (0-5).** Red line is a LOESS curve.


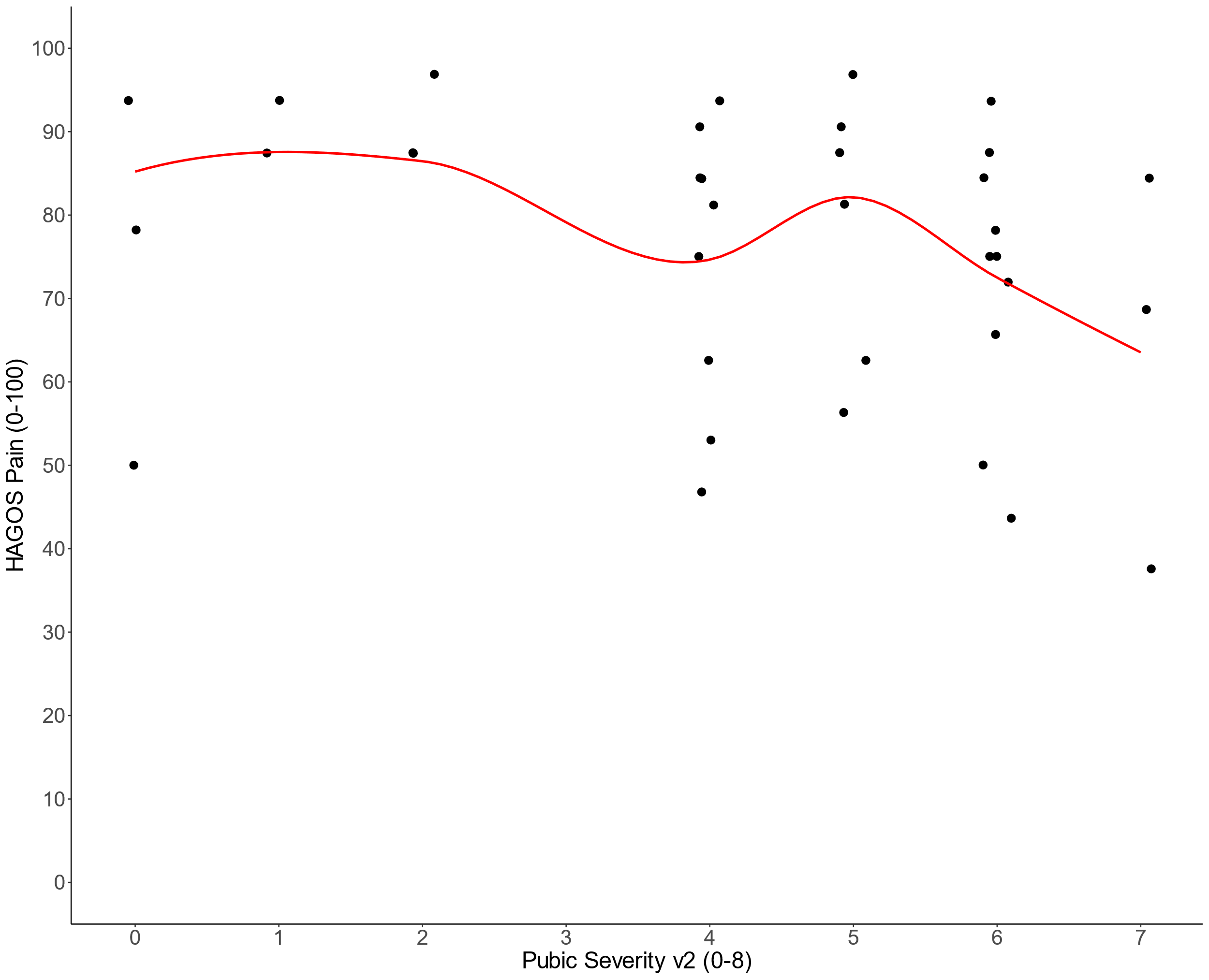


**Figure A6 Scatterplot of HAGOS Pain and PSRS Score 2 (0-8).** Red line is a LOESS curve.


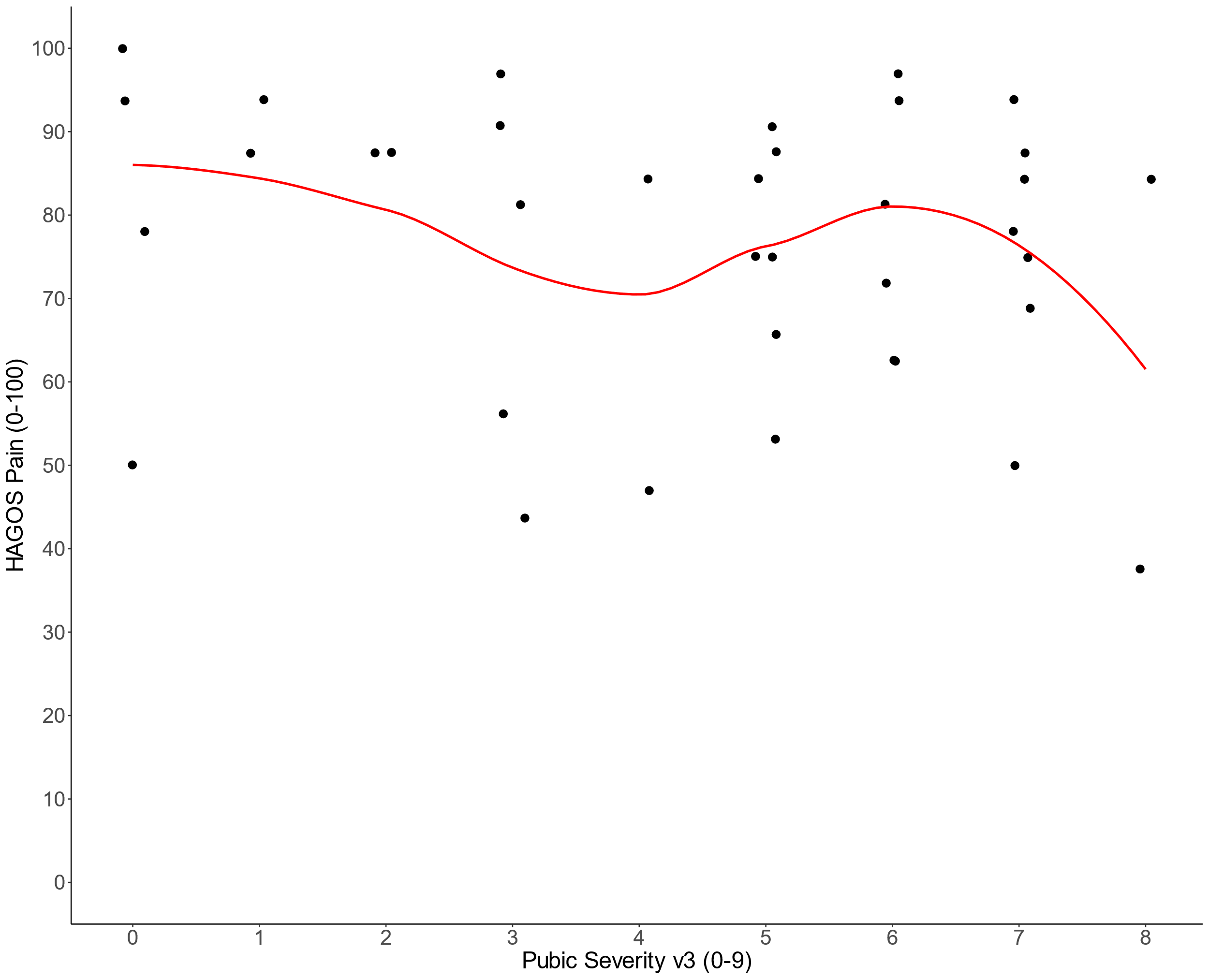


**Figure A7 Scatterplot of HAGOS Pain and PSRS Score 3 (0-10).** Red line is a LOESS curve.


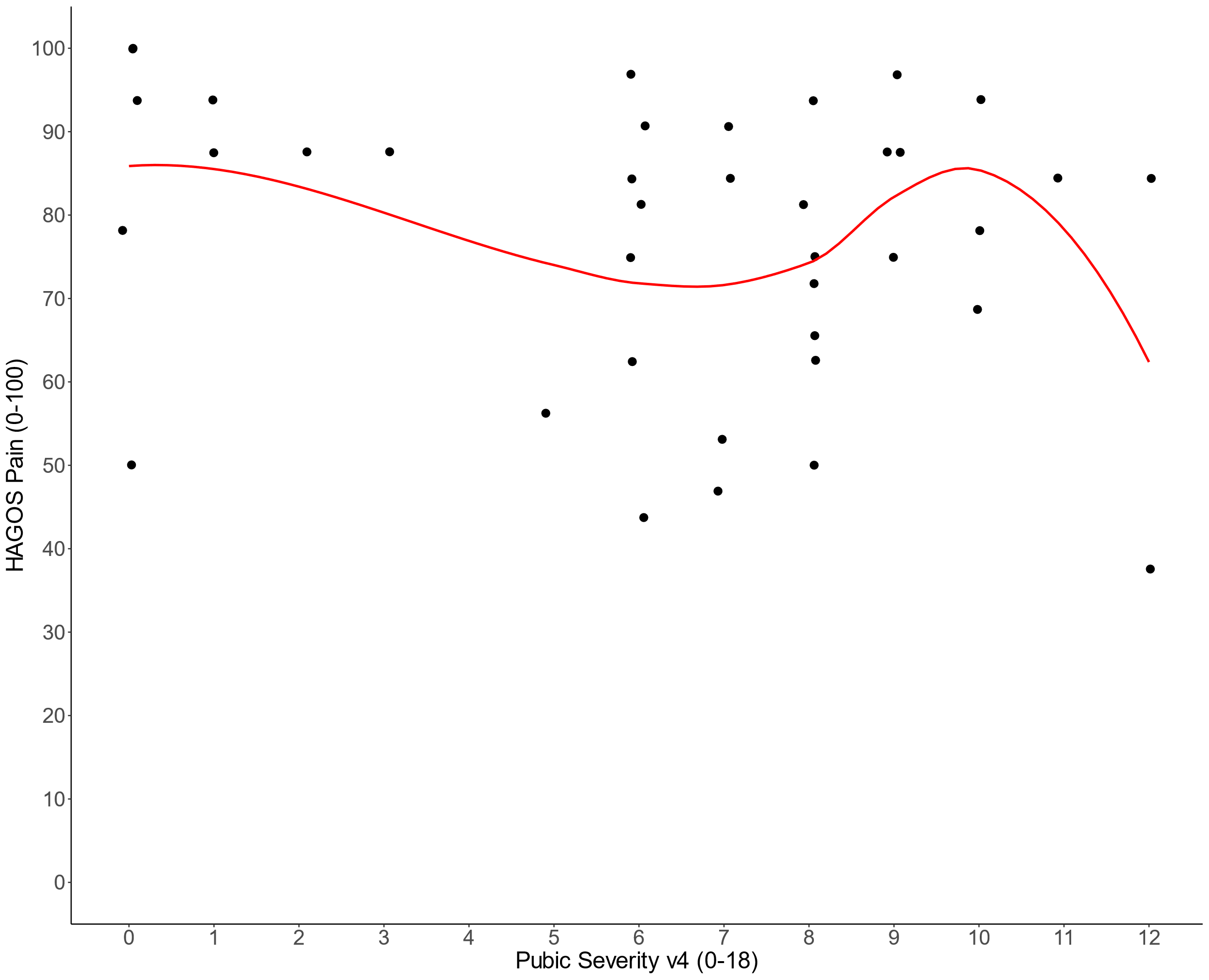


**Figure A8 Scatterplot of HAGOS Pain and PSRS Score 4 (0-18).** Red line is a LOESS curve.


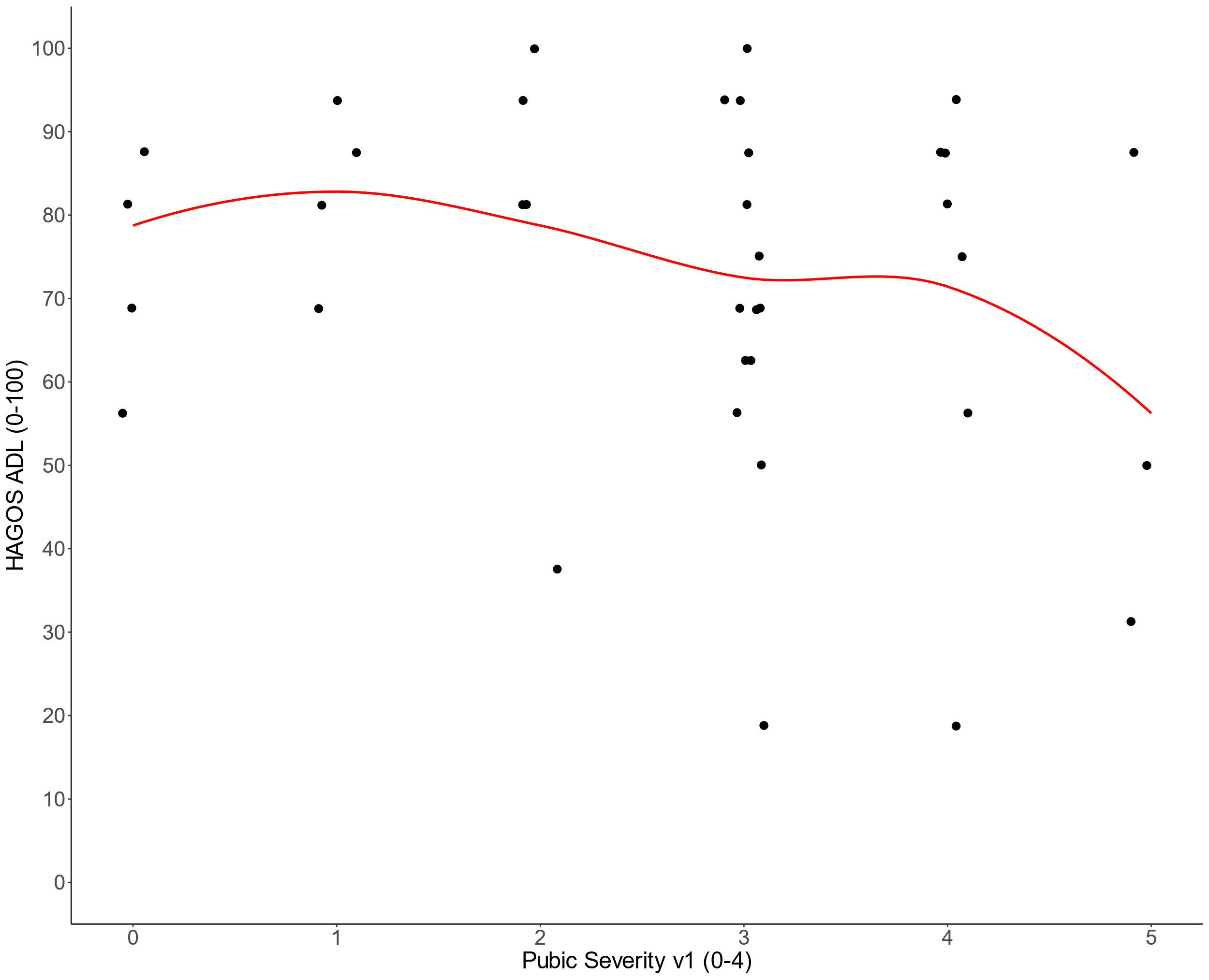


**Figure A9 Scatterplot of HAGOS ADL and PSRS Score 1 (0-5).** Red line is a LOESS curve.


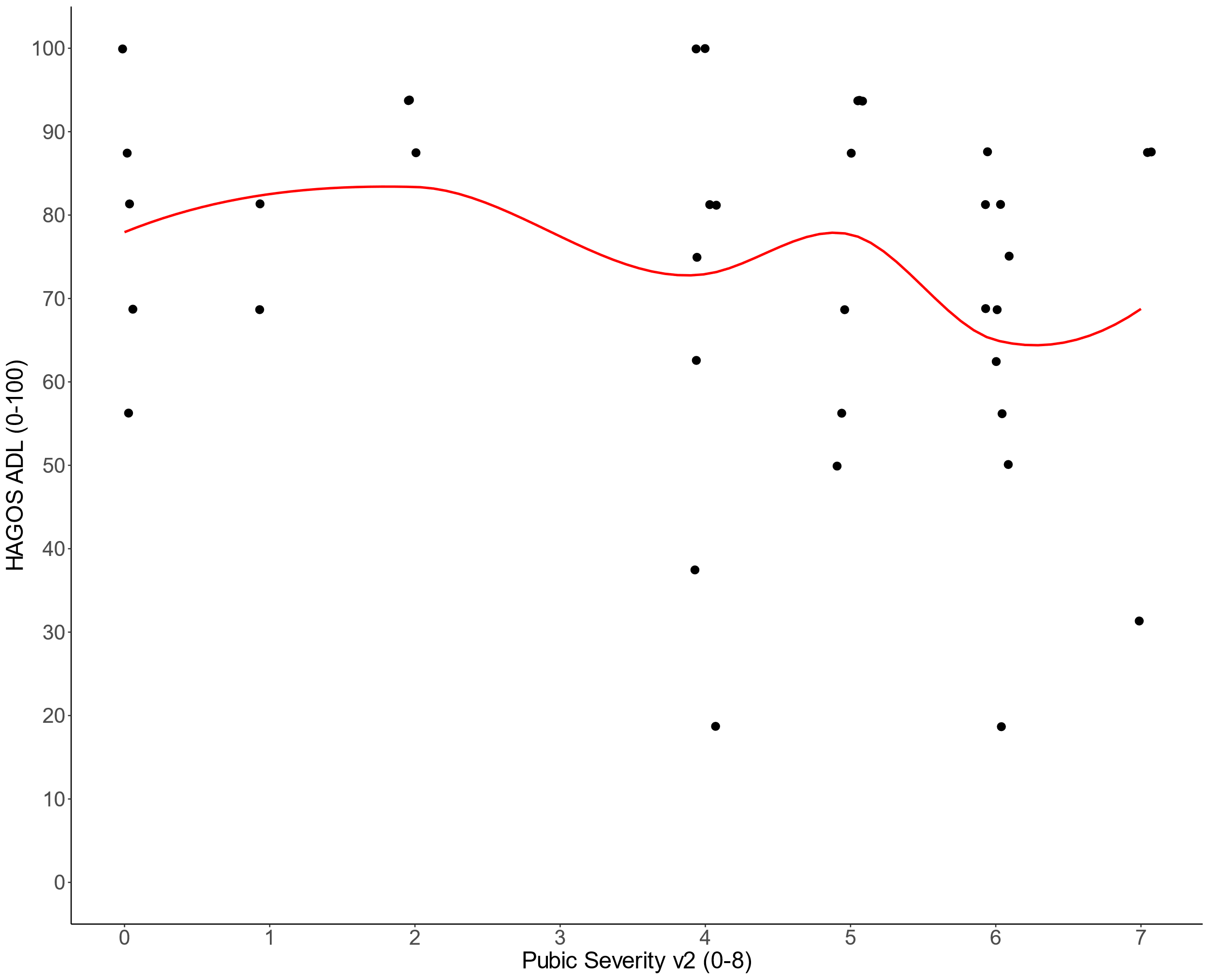


**Figure A10 Scatterplot of HAGOS ADL and PSRS Score 2 (0-8).** Red line is a LOESS curve.


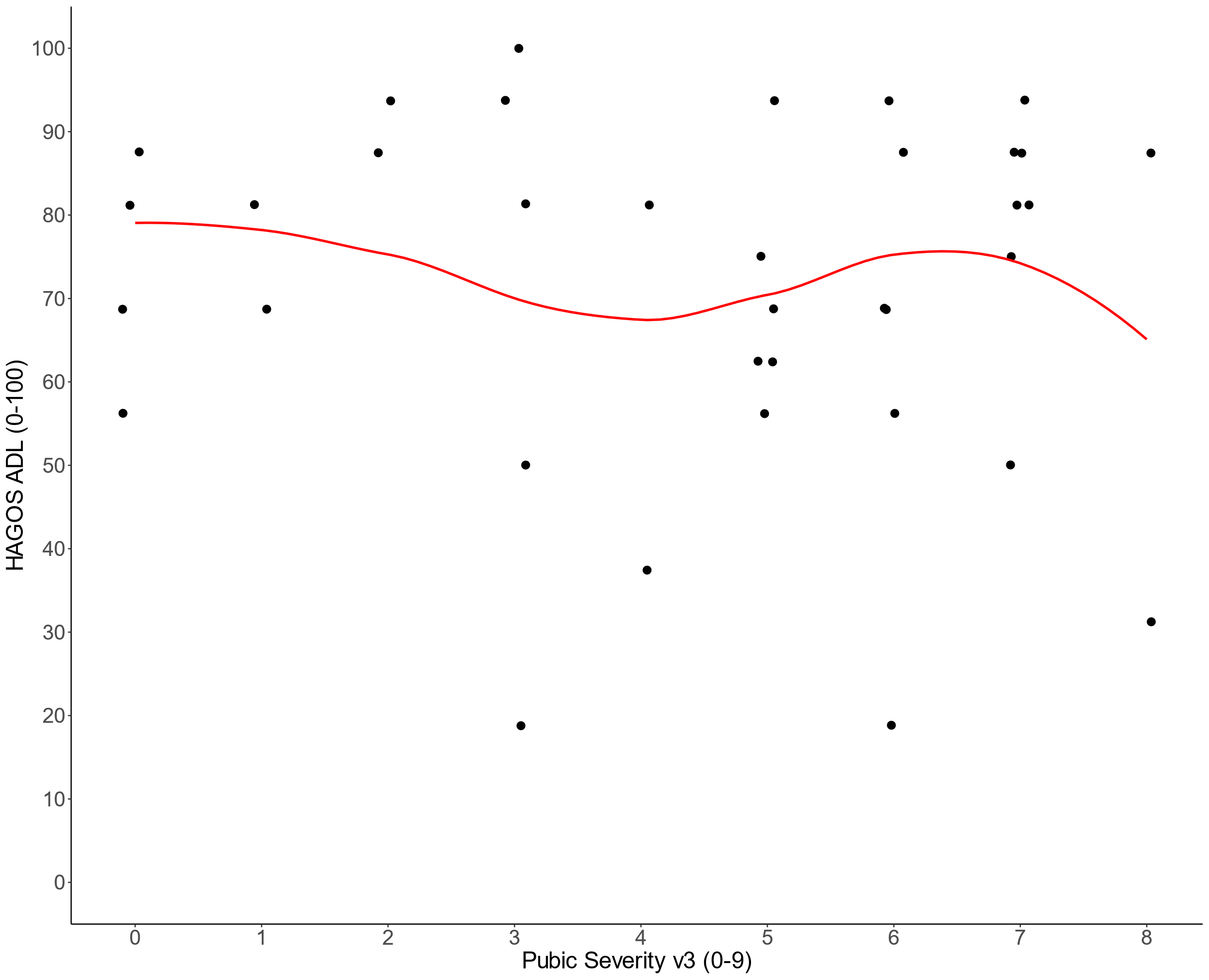


**Figure A11 Scatterplot of HAGOS ADL and PSRS Score 3 (0-10).** Red line is a LOESS curve.


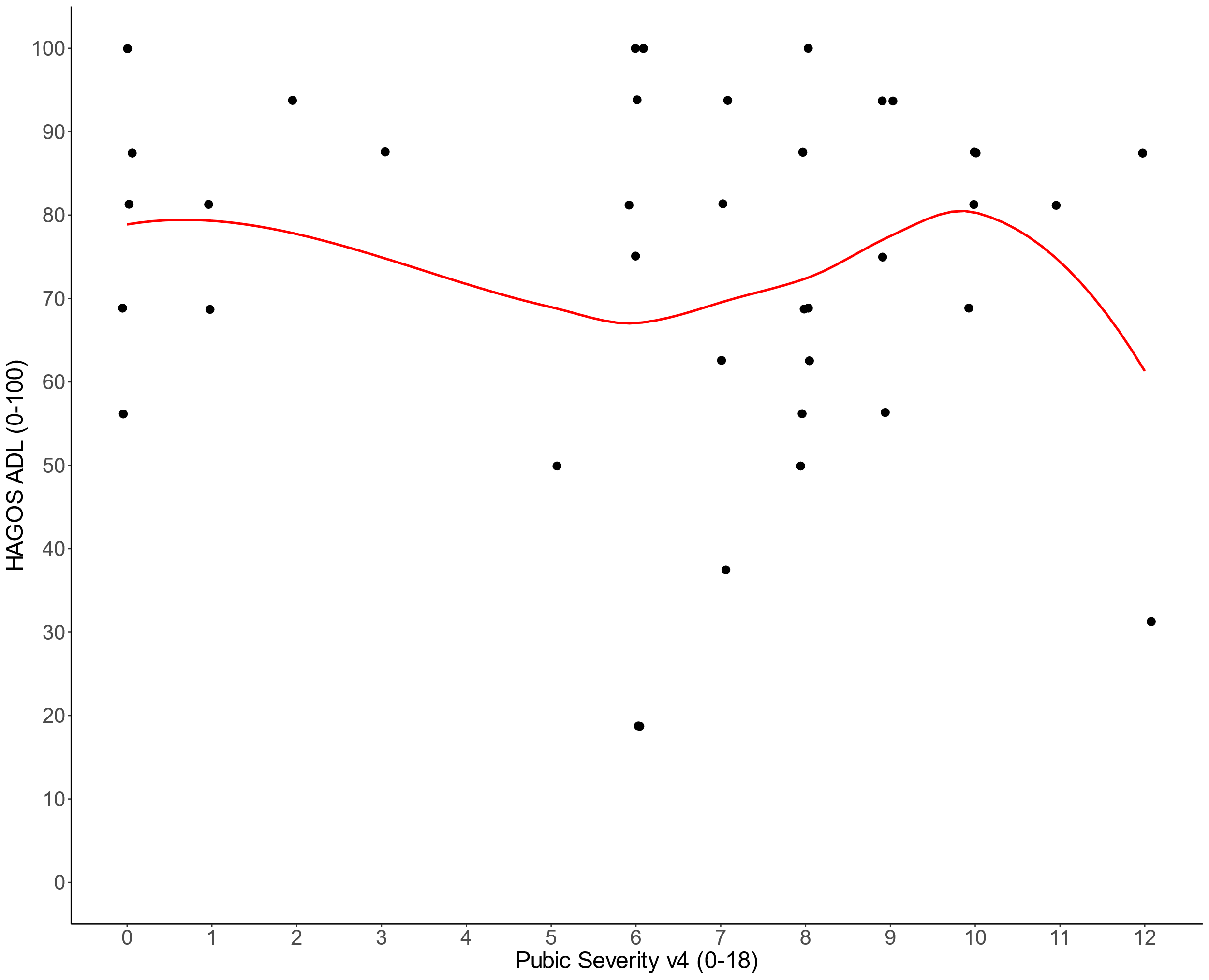


**Figure A12 Scatterplot of HAGOS ADL and PSRS Score 4 (0-18).** Red line is a LOESS curve.


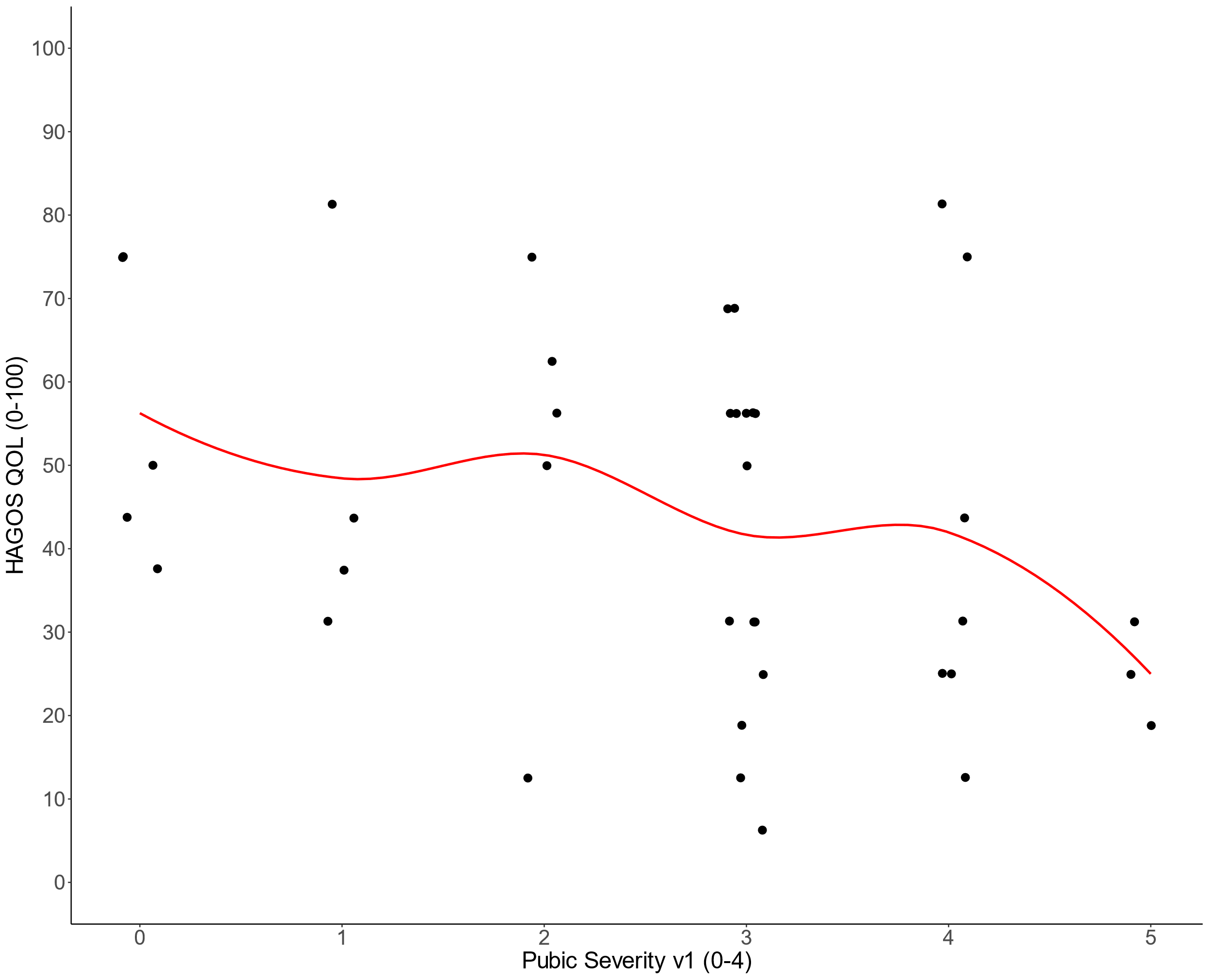


**Figure A13 Scatterplots of HAGOS Quality of Life and PSRS Score 1 (0-5).** Red line is a LOESS curve.


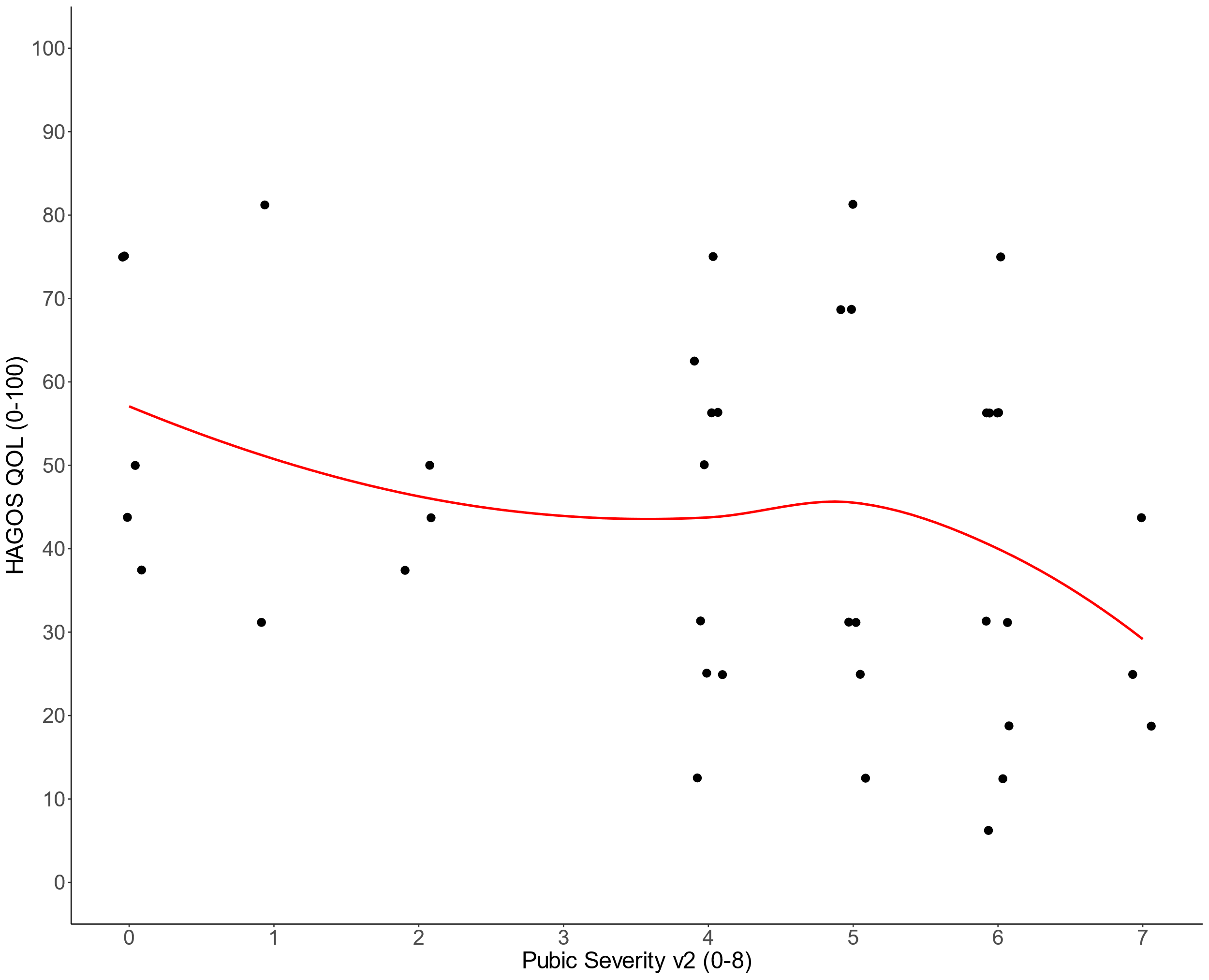


**Figure A14 Scatterplots of HAGOS Quality of Life and PSRS Score 2 (0-8).** Red line is a LOESS curve.


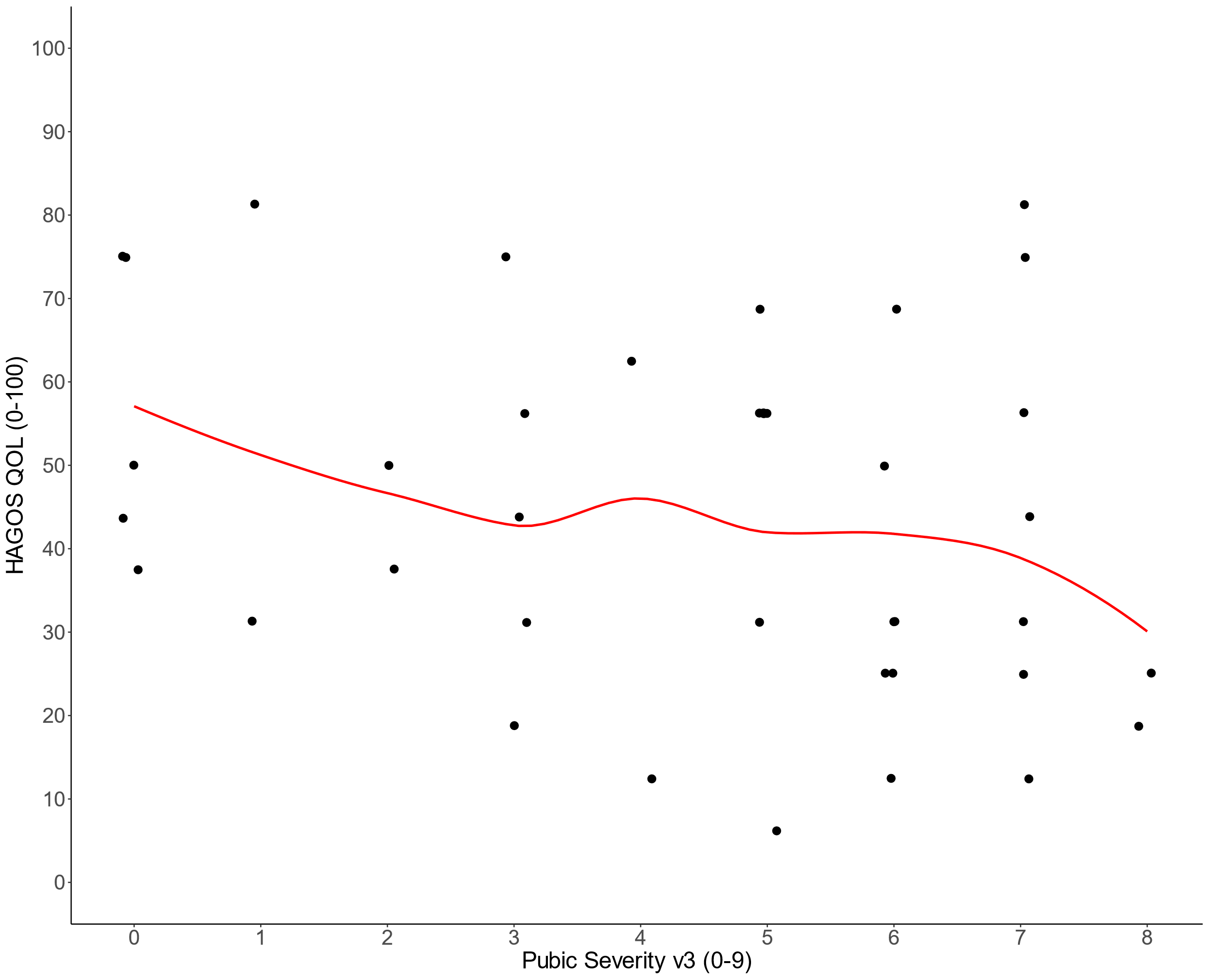


**Figure A15 Scatterplots of HAGOS Quality of Life and PSRS Score 3 (0-10).** Red line is a LOESS curve.


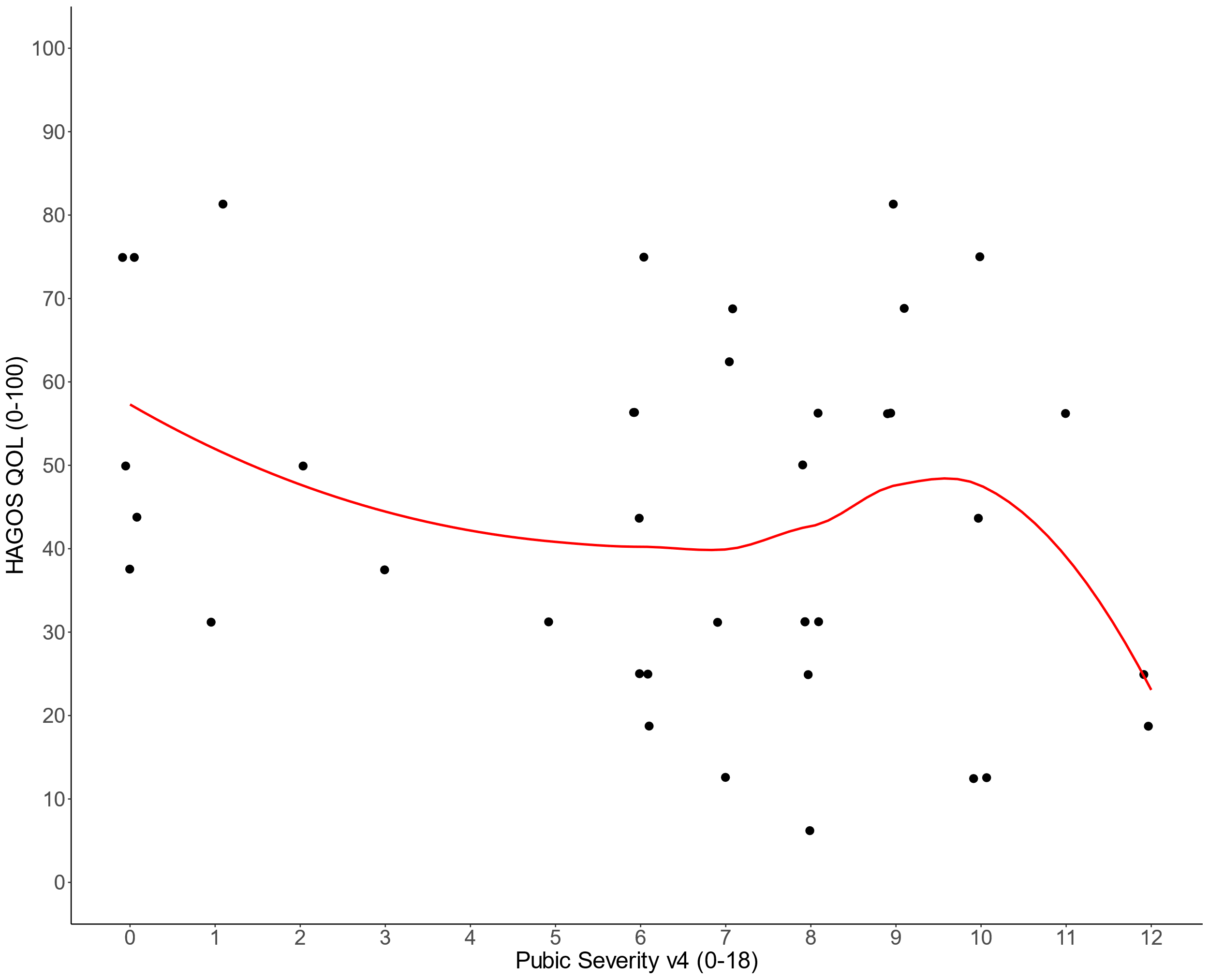


**Figure A16 Scatterplots of HAGOS Quality of Life and PSRS Score 4 (0-18).** Red line is a LOESS curve.

1. **Linear regression models for associations between Pubic Symphysis Radiographic Severity (PSRS) Scores and 5SST and HAGOS Subscales in the symptomatic football players (n=39)**

| **Table A5 Associations between Pubic Symphysis Radiographic Severity (PSRS) Scores and 5SST** | | | | |
| --- | --- | --- | --- | --- |
|  | **5SST** | | | |
|  | *Coef.* | *(95% CI)* | *p-value* | *adj. p* |
| Intercept | 7.8 | (5.5 to 10.1) | < 0.00 |  |
| Bone Lucency | **–3.9** | **(–7.0 to –0.8)** | **0.02** | 0.12 |
| Proliferation | 2.3 | (–0.0 to 4.5) | 0.06 | 0.12 |
| Fragmentation | 0.3 | (–2.2 to 2.7) | 0.82 | 0.99 |
| Sclerosis | 0.5 | (–1.7 to 2.6) | 0.69 | 0.99 |
| Narrow Pubic Joint space width | **–2.2** | **(–4.2 to –0.2)** | **0.04** | 0.12 |
| *Coef. = beta coefficient, CI = confidence interval, adj. p = Benjamini-Hocberg adjusted p-value* | | | | |

| **Table A6** **Associations between Pubic Symphysis Radiographic Severity (PSRS) Scores and HAGOS Subscales** | | | | | | | | | | | | |
| --- | --- | --- | --- | --- | --- | --- | --- | --- | --- | --- | --- | --- |
|  | **HAGOS Pain** | | | | **HAGOS Symptoms** | | | | **HAGOS ADL** | | | |
|  | *Coef.* | *(95% CI)* | *p-value* | *adj. p* | *Coef.* | *(95% CI)* | *p-value* | *adj. p* | *Coef.* | *(95% CI)* | *p-value* | *adj. p* |
| Intercept | 84.4 | (70.8 to 97.9) | < 0.00 |  | 59.2 | (44.0 to 74.4) | <0.00 |  | 78.8 | ( 60.2 to 97.3) | < 0.00 |  |
| Bone Lucency | 5.4 | (–13.0 to 23.8) | 0.57 | 0.68 | 9.6 | (–11.1 to 30.2) | 0.37 | 0.53 | 6.1 | (–19.0 to 31.3) | 0.64 | 0.86 |
| Proliferation | 7.4 | (–6.2 to 20.9) | 0.29 | 0.59 | 6.1 | (–9.1 to 21.2) | 0.44 | 0.53 | 7.4 | (–11.1 to 25.9) | 0.44 | 0.86 |
| Fragmentation | –11.1 | (–25.5 to 3.4) | 0.14 | 0.43 | –9.0 | (–25.2 to 7.3) | 0.29 | 0.53 | –8.3 | (–28.2 to 11.5) | 0.42 | 0.86 |
| Sclerosis | **–20.8** | **(–33.7 to –7.9)** | **< 0.00** | **0.02** | –12.0 | (–26.5 to 2.5) | 0.11 | 0.53 | **–20.6** | **(–38.2 to –2.9)** | **0.03** | 0.17 |
| Narrow Pubic Joint space width | –3.8 | (–15.7 to 8.1) | 0.53 | 0.68 | –6.0 | (–19.3 to 7.4) | 0.39 | 0.53 | –3.1 | (–19.3 to 13.2) | 0.72 | 0.86 |
| *Coef. = beta coefficient. CI = confidence interval. adj. p = Benjamini-Hocberg adjusted p-value* | | | | | | | | | | | | |

| **Table A6 continued** | | | | |  |  |  | |  |  |  | |
| --- | --- | --- | --- | --- | --- | --- | --- | --- | --- | --- | --- | --- |
|  | **HAGOS Sport** | | | | **HAGOS PA** | | | | **HAGOS QoL** | | | |
|  | *Coef.* | *(95% CI)* | *p-value* | *adj. p* | *Coef.* | *(95% CI)* | *p-value* | *adj. p* | *Coef.* | *(95% CI)* | *p-value* | *adj. p* |
| Intercept | 45.0 | (28.9 to 61.1) | < 0.00 |  | 37.5 | (12.3 to 62.7) | < 0.00 | 0.04 | 56.3 | (38.0 to 74.5) | < 0.00 |  |
| Bone Lucency | 11.5 | (–10.3 to 33.4) | 0.31 | 0.61 | –0.8 | (–35.0 to 33.4) | 0.96 | 0.96 | –8.5 | (–33.3 to 16.3) | 0.51 | 1.00 |
| Proliferation | 5.9 | (–10.1 to 22.0) | 0.47 | 0.71 | 5.3 | (–19.9 to 30.4) | 0.68 | 0.96 | 0.1 | (–18.1 to 18.3) | 0.99 | 1.00 |
| Fragmentation | –16.4 | (–33.6 to 0.8) | 0.07 | 0.21 | –22.8 | (–49.7 to 4.2) | 0.11 | 0.32 | –18.9 | (–38.4 to 0.7) | 0.07 | 0.40 |
| Sclerosis | –14.8 | (–30.1 to 0.5) | 0.07 | 0.21 | 4.4 | (–19.6 to 28.3) | 0.72 | 0.96 | –0.5 | (–17.8 to 16.9) | 0.96 | 1.00 |
| Narrow Pubic Joint space width | 2.5 | (–11.6 to 16.6) | 0.73 | 0.87 | 1.7 | (–20.4 to 23.8) | 0.88 | 0.96 | –5.4 | (–21.4 to 10.6) | 0.51 | 1.00 |
|  |  |  |  | |  |  |  | |  |  |  | |

1. **Post-hoc analyses including only symptomatic football players with adductor- or pubic-related groin pain.**

| **Table A7 Prevalence of pubic-related findings on side- and person level including only symptomatic football players with adductor- or pubic-related groin pain** | | | | | | | | | |
| --- | --- | --- | --- | --- | --- | --- | --- | --- | --- |
|  | **Symptomatic**  **football players** | | | **Asymptomatic**  **football players** | | | **Asymptomatic**  **non-football athletes** | | |
| **Pubic-related radiographic finding, n (%)** | *Right,*  *n = 26* | *Left,*  *n = 26* | *Person,*  *n = 26* | *Right,*  *n = 18* | *Left,*  *n = 18* | *Person,*  *n = 18* | *Right,*  *n = 20* | *Left,*  *n = 20* | *Person,*  *n = 20* |
| **Bone Lucency** | 20 (77%)* | 20 (77%)* | 21 (81%)** | 11 (61%)* | 12 (67%)* | 15 (83%)** | 8 (40%)* | 7 (35%)* | 8 (40%)** |
| *Erosion-Like Configuration* | 20 (77%)* | 20 (77%)* | 21 (81%)** | 11 (61%)* | 12 (67%)* | 15 (83%)** | 8 (40%)* | 7 (35%)* | 8 (40%)** |
| Superior/Central ELC | 15 (58%) | 18 (69%) | 19 (73%) | 8 (44%) | 10 (56%) | 10 (56%) | 6 (30%) | 7 (35%) | 8 (40%) |
| Inferior ELC | 17 (65%)* | 19 (73%)* | 20 (77%)** | 10 (56%)* | 11 (61%)* | 14 (78%)** | 5 (25%)* | 6 (30%)* | 6 (30%)** |
| *Cysts* | 4 (15%) | 7 (27%) | 9 (35%) | 2 (11%) | 1 (6%) | 2 (11%) | 0 (0%) | 2 (10%) | 2 (10%) |
| **Proliferation** | 14 (54%)* | 14 (54%)* | 16 (61%)* | 11 (61%)* | 10 (56%)* | 11 (61%)* | 4 (20%)* | 4 (20%)* | 5 (25%)* |
| *Superior Proliferation* | 12 (46%)* | 11 (42%) | 14 (54%)* | 10 (56%)* | 7 (39%) | 10 (56%)* | 3 (15%)* | 3 (15%) | 4 (20%)* |
| *Central Proliferation* | 4 (15%) | 6 (23%) | 9 (35%)* | 6 (33%) | 5 (28%) | 9 (50%)* | 1 (5%) | 1 (5%) | 2 (10%)* |
| *Inferior Proliferation* | 5 (19%) | 4 (15%) | 9 (35%)* | 5 (28%) | 4 (22%) | 6 (33%)* | 1 (5%) | 0 (0%) | 1 (5%)* |
| **Fragmentations** | 1 (4%) | 2 (8%) | 3 (11%) | 0 (0%) | 1 (6%) | 1 (6%) | 0 (0%) | 0 (0%) | 0 (0%) |
| *Central Fragmentation* | 0 (0%) | 0 (0%) | 0 (0%) | 0 (0%) | 1 (6%) | 1 (6%) | 0 (0%) | 0 (0%) | 0 (0%) |
| *Inferior Fragmentation* | 1 (4%) | 2 (8%) | 3 (11%) | 0 (0%) | 0 (0%) | 0 (0%) | 0 (0%) | 0 (0%) | 0 (0%) |
| **Sclerosis** | 16 (61%)** | 18 (69%)** | 18 (69%)** | 8 (44%)** | 9 (50%)** | 9 (50%)** | 2 (10%)** | 3 (15%)** | 3 (15%)** |
| **Pubic Joint Space width**, mm, mean (SD) |  |  | 3 (1) |  |  | 3 (1) |  |  | 3 (1) |
| Narrow pubic joint space width |  |  | 6 (23) |  |  | 3 (17) |  |  | 4 (20) |
| **χ^2^-test p-value < 0.05 indicating statistically significantly difference across all three groups.*  ***χ^2^-test p-value < 0.005 indicating statistically significantly difference across all three groups.* | | | | | | | | | |

| **Table A8 Pairwise comparison of pubic-related findings on person-level including only symptomatic football players with adductor- or pubic-related groin pain** | | | | | | | | |
| --- | --- | --- | --- | --- | --- | --- | --- | --- |
|  | **Symptomatic football players (n = 26)**  **vs**  **Asymptomatic football players (n=18)** | | | | **Symptomatic football players (n = 26)**  **vs**  **Asymptomatic non-football athletes (n=20)** | | | |
| **Pubic-related findings** | ***OR*** | ***(95% CI)*** | ***p-value*** | ***Adj. p*** | ***OR*** | ***(95% CI)*** | ***p-value*** | ***Adj. p*** |
| **Bone Lucency** | 1.19 | (0.19-8.83) | 1.00 | 1.00 | **0.17** | **(0.03-0.70)** | **0.01** | **0.04** |
| *Erosion-Like Configuration* | 1.19 | (0.19-8.83) | 1.00 | 1.00 | **0.17** | **(0.03-0.70)** | **0.01** | **0.04** |
| Superior/Central ELC | 0.47 | (0.11-1.97) | 0.33 | 0.58 | **0.25** | **(0.06-1.00)** | **0.04** | 0.12 |
| Inferior ELC | 1.05 | (0.20-6.04) | 1.00 | 1.00 | **0.14** | **(0.03-0.57)** | **< 0.00** | **0.03** |
| *Cysts* | 0.24 | (0.02-1.44) | 0.15 | 0.39 | 0.22 | (0.02-1.27) | 0.08 | 0.23 |
| **Proliferation** | 0.98 | (0.24-4.07) | 1.00 | 1.00 | **0.22** | **(0.05-0.87)** | **0.02** | 0.10 |
| *Superior Proliferation* | 1.07 | (0.27-4.26) | 1.00 | 1.00 | **0.22** | **(0.04-0.95)** | **0.03** | 0.12 |
| *Central Proliferation* | 1.86 | (0.47-7.65) | 0.36 | 0.60 | 0.22 | (0.02-1.27) | 0.08 | 0.23 |
| *Inferior Proliferation* | 0.95 | (0.21-3.97) | 1.00 | 1.00 | **0.10** | **(0.00-0.88)** | **0.03** | 0.12 |
| **Fragmentations** | 0.46 | (0.01-6.30) | 0.63 | 0.93 | 0.00 | (0.00-3.10) | 0.25 | 0.48 |
| *Central Fragmentation* | - | - | - | - | - | - | - | - |
| *Inferior Fragmentation* | 0.00 | (0.00-3.46) | 0.26 | 0.48 | 0.00 | (0.00-3.10) | 0.25 | 0.48 |
| **Sclerosis** | 0.45 | (0.11-1.83) | 0.22 | 0.48 | **0.08** | **(0.01-0.40)** | **< 0.00** | **0.01** |
| **Narrow Pubic Joint Space** | 0.67 | (0.09-3.80) | 0.72 | 1.00 | 0.84 | (0.15-4.26) | 1.00 | 1.00 |
| *Interpretation: a lower odds ratio indicates a lower risk of findings in the asymptomatic group. OR = Odds ratio. CI = Confidence Interval.* | | | | | | | | |

1. **Table with prevalence estimates, un- and adjusted p-values**

| **Table A9 Pubic-related radiographic finding prevalences with un- and adjusted p-values for Chi^2^ test across all groups.** | | | | | | | | | |  |  |  |
| --- | --- | --- | --- | --- | --- | --- | --- | --- | --- | --- | --- | --- |
|  | **Symptomatic**  **football players** | | | **Asymptomatic**  **football players** | | | **Asymptomatic**  **non-football athletes** | | | **P-values from Chi2 test for prevalence**  **differences across three groups** | | |
|  | *Right*  *n =* | *Left*  *n =* | *Person*  *n = 39* | *Right*  *n = 18* | *Left*  *n = 18* | *Person*  *n = 18* | *Right*  *n = 20* | *Left*  *n = 20* | *Person*  *n = 20* | *Right*  *p-value, adj. p* | *Left*  *p-value, adj. p* | *Person*  *p-value, adj. p* |
| **Bone Lucency** | 33 (85%) | **30 (77%)** | **34 (87%)** | 11 (61%) | **12 (67%)** | **15 (83%)** | 8 (40%) | **7 (35%)** | **8 (40%)** | ***0.002, 0.02*** | ***0.006, 0.02*** | ***0.0003, 0.002*** |
| *Erosion-Like Configuration* | 33 (85%) | **30 (77%)** | **34 (87%)** | 11 (61%) | **12 (67%)** | **15 (83%)** | 8 (40%) | **7 (35%)** | **8 (40%)** | ***0.002, 0.02*** | ***0.006, 0.02*** | ***0.0003, 0.002*** |
| Superior/Central ELC | 26 (67%) | 27 (69%) | **30 (77%)** | 8 (44%) | 10 (56%) | **10 (56%)** | 6 (30%) | 7 (35%) | **8 (40%)** | ***0.022****, 0.05* | ***0.042****, 0.08* | ***0.0171, 0.03*** |
| Inferior ELC | **27 (69%)** | 26 (67%) | **31 (80%)** | **10 (56%)** | 11 (61%) | **14 (78%)** | **5 (25%)** | 6 (30%) | **6 (30%)** | ***0.005, 0.02*** | ***0.024****, 0.05* | ***0.0004, 0.002*** |
| *Cysts* | 5 (13%) | 10 (26%) | 12 (31%) | 2 (11%) | 1 (6%) | 2 (11%) | 0 (0%) | 2 (10%) | 2 (10%) | *0.253, 0.31* | *0.108, 0.16* | *0.0908, 0.14* |
| **Proliferation** | **22 (56%)** | 22 (56%) | **26 (67%)** | **11 (61%)** | 10 (56%) | **11 (61%)** | **4 (20%)** | 4 (20%) | **5 (25%)** | ***0.013, 0.04*** | ***0.021****, 0.05* | ***0.0080, 0.02*** |
| *Superior Proliferation* | **20 (51%)** | 18 (46%) | **23 (59%)** | **10 (56%)** | 7 (39%) | **10 (56%)** | **3 (15%)** | 3 (15%) | **4 (20%)** | ***0.013, 0.04*** | *0.061, 0.10* | ***0.0137, 0.03*** |
| *Central Proliferation* | 8 (20%) | 12 (31%) | **16 (41%)** | 6 (33%) | 5 (28%) | **9 (50%)** | 1 (5%) | 1 (5%) | **2 (10%)** | *0.086, 0.13* | *0.076, 0.12* | ***0.0193, 0.03*** |
| *Inferior Proliferation* | 6 (15%) | 10 (26%) | **16 (41%)** | 5 (28%) | 4 (22%) | **6 (33%)** | 1 (5%) | 0 (0%) | **1 (5%)** | *0.154, 0.21* | ***0.047****, 0.09* | ***0.0156, 0.03*** |
| **Fragmentations** | 2 (5%) | 5 (13%) | 6 (15%) | 0 (0%) | 1 (6%) | 1 (6%) | 0 (0%) | 0 (0%) | 0 (0%) | *0.368, 0.40* | *0.203, 0.26* | *0.1261, 0.16* |
| *Central Fragmentation* | 1 (3%) | 3 (8%) | 3 (8%) | 0 (0%) | 1 (6%) | 1 (6%) | 0 (0%) | 0 (0%) | 0 (0%) | *0.610, 0.61* | *0.451, 0.47* | *0.4505, 0.52* |
| *Inferior Fragmentation* | 2 (5%) | 2 (5%) | 4 (10%) | 0 (0%) | 0 (0%) | 0 (0%) | 0 (0%) | 0 (0%) | 0 (0%) | *0.368, 0.40* | *0.368, 0.40* | *0.1280, 0.16* |
| **Sclerosis** | **23 (59%)** | **23 (60%)** | **25 (64%)** | **8 (44%)** | **9 (50%)** | **9 (50%)** | **2 (10%)** | **3 (15%)** | **3 (15%)** | ***0.002, 0.02*** | ***0.005, 0.02*** | ***0.0017, 0.006*** |
| **Pubic Joint Space width**,  mm, mean (SD) | - | - | 3 (1) | - | - | 3 (1) | - | - | 3 (1) |  |  | *0.5806, 0.62* |
| Narrow pubic joint space width | - | - | 11 (28**%**) | - | - | 3 (17**%**) | - | - | 4 (20**%**) |  |  | *0.5534, 0.58* |
|  | | | | | | | | | |  |  |  |
