# Supplementary material for: Pubic‐Related Radiographic Findings in Male Football Players With Long‐Standing Groin Pain, and Asymptomatic Controls — Are They Clinically Relevant?
Source: Scand J Med Sci Sports. 2025 May 13;35(5):e70068. doi: 10.1111/sms.70068 (PMC12070245; doi:10.1111/sms.70068)
Supplement: Supplementary file 2 — Data S1. [file SMS-35-e70068-s001.docx]

**Supplementary file**

**Content:**

1. Description of radiographic procedure
2. Descriptive radiographic hip-related findings
3. The Copenhagen five-second squeeze test
4. Clinical examination pain provocation tests
5. Clinical entities of groin pain
6. **Description of Radiographic Procedure**

All participants underwent conventional radiographic examination of the hip and pelvis including a cross-table lateral hip view radiograph of each hip and a standing anteroposterior pelvic view radiograph (see the supplementary file for detailed description of patient positioning). Radiographs were accessed in the RIS/PACS system, Agfa IMPAX Client (Bundle 5) (Agfa-Gevaert Group, Belgium), and the surgical planning software mediCAD (MediCAD Hectec GmbH, Germany). Two musculoskeletal radiologists (SB and TT), with 11 and 25 years of experience in hip-related radiographic assessment, rated the radiographs independently and in random order, blinded to previously collected data. Both radiologists had no prior experience with the standardized protocol for evaluating pubic-related parameters. The new ratings was collected using REDCap ^1^. The radiologists were trained and calibrated on all radiographic parameters by PH, an orthopedic surgeon and author of the Aspetar protocol ^2^. Rater 2 (TT) rated all radiographs twice with an 8-week interval. Hip- and pubic-related parameters are illustrated in Figures 3a-3f and 4a-4e, respectively.

Pubic-related parameters, ischial spine sign, posterior wall sign, and crossover sign were rated 21 weeks before the alpha angle, lateral center-edge angle, acetabular index angle, and hip joint space width due to technical issues accessing mediCAD. Furthermore, Rater 1 identified a technical error when measuring the lateral center-edge angle in mediCAD - one reference line was not calibrated correctly to the radiograph orientation. As a results, Rater 2 had to re-measure the lateral center-edge angle in 57 participants. To assess the radiographic integrity, each radiologist screened for pelvic tilt by measured the distance from the sacrococcygeal joint to the upper rim of the pubic bones/joint, excluding radiographs if the distance < 25 or > 55 mm. Radiographs were also excluded if the gonad shields covered the symphysis joint or inferior rami of the pubic bone, or if the radiologists judged that the positioning or quality issues would influence parameter accuracy.

1. **Descriptive radiographic hip-related findings**

Participants were classified with CAM (alpha angle >60°^3,4^), pincer (LCEA ≥ 40°^4^ OR LCEA ≥ 35° and a acetabular index angle < 0°^4^), borderline hip dysplasia (LCEA 20-25°) and/or hip dysplasia (a LCEA < 20° or Acetabular index angle > 13°^5^).

**Alpha-Angle.**

**Definition:** The angle between 1) a line from the center of the femoral neck to the center of the femoral head, and 2) a line from the center of the femoral head to the point where the femoral head-neck junction extends beyond the margin of the circle ^3^.

**View**: Cross-table lateral hip.

**Defines:** *Cam morphology* as alpha angle ≥ 60° ^3^.


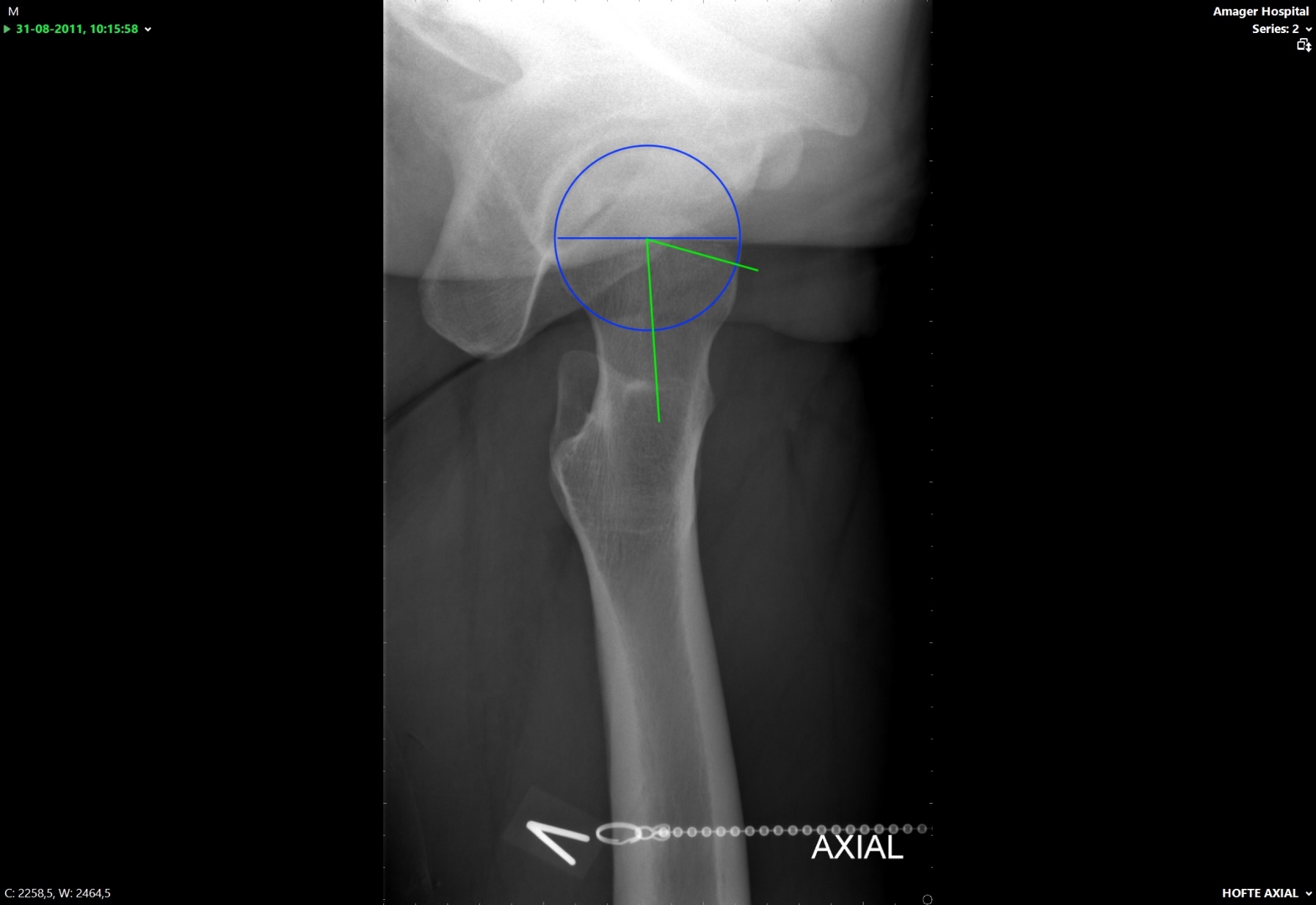


**Lateral Center Edge Angle.**

**Definition:** The angle between 1) a vertical line through the femoral head center and 2) a line between the femoral head center and the lateral sourcil of the acetabulum, as defined by Wiberg ^3^.

**View**: Anteroposterior.

**Defines:** *Pincer morphology* as 1) a lateral center edge angle ≥ 40° OR 2) a lateral Center Edge angle ≥ 35° AND Acetabular index < 0° ^3^.

*Hip dysplasia* as 1) lateral center edge angle ≤ 25


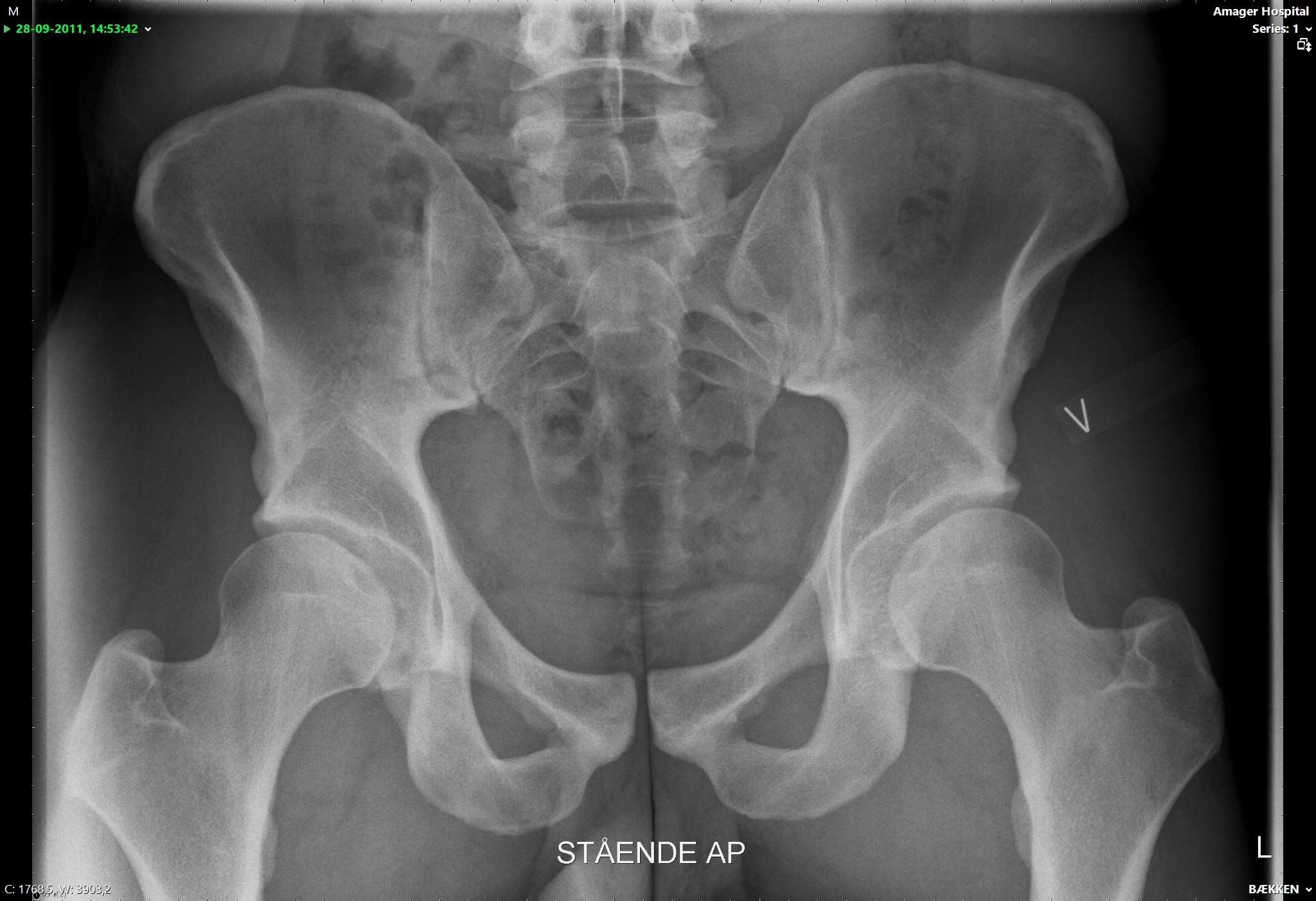


**Acetabular Index Angle.**

**Definition:** The angle between a horizontal line and a line through the most medial point of the sclerotic zone of the acetabulum and the sourcil ^3^.

**View**: Anteroposterior.

**Defines:** *Pincer morphology* as 2) a lateral Center Edge angle ≥ 35° AND Acetabular index < 0° ^3^.

*Hip dysplasia* as 2) acetabular index > 13° ^3^.


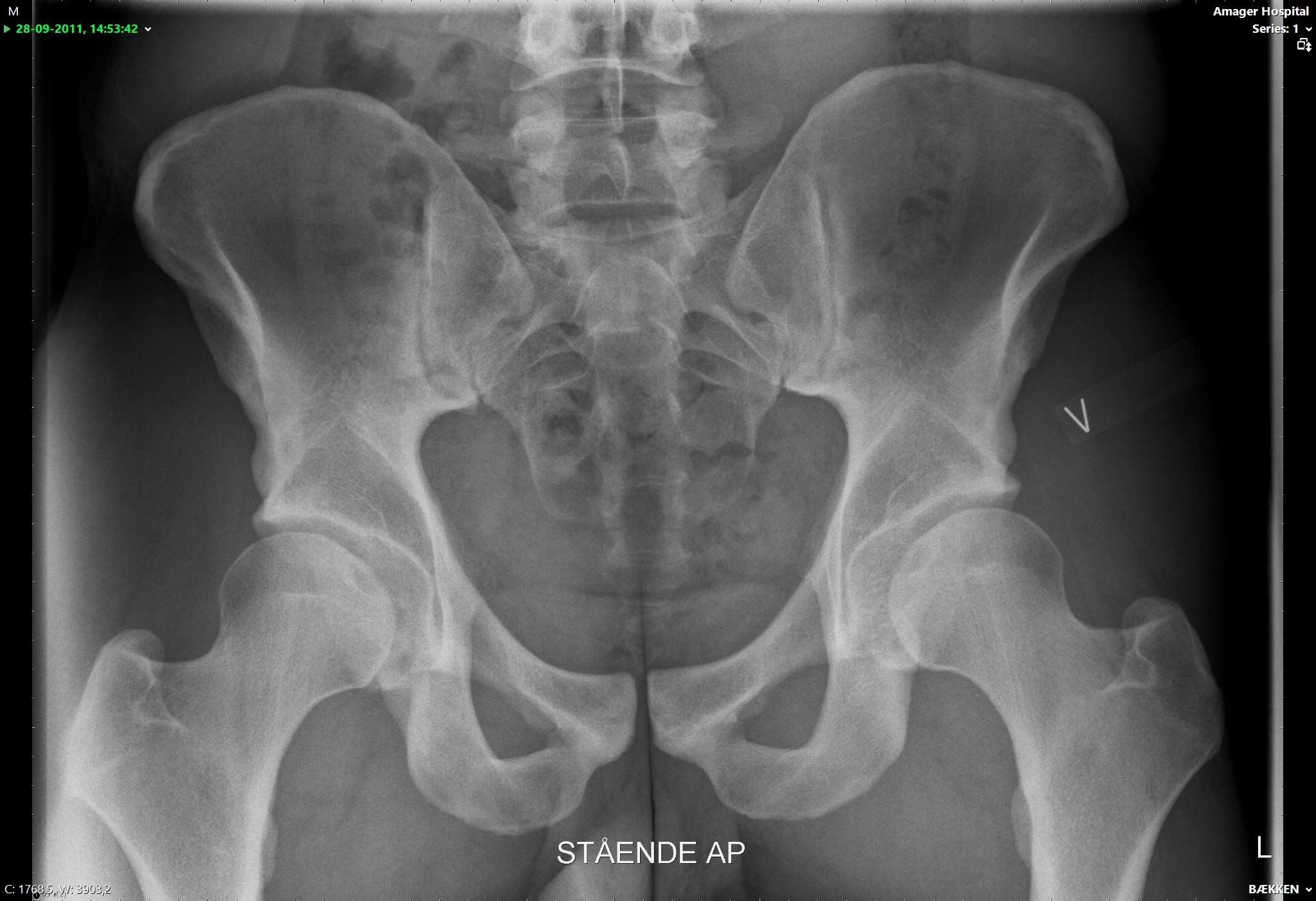


**Hip Joint Space Width.**

**Definition:** The distance between the femoral head and the lateral sourcil at the acetabulum ^3^.

**View**: Anteroposterior.

**Defines:** *Joint space narrowing* as hip joint space width < 3mm.


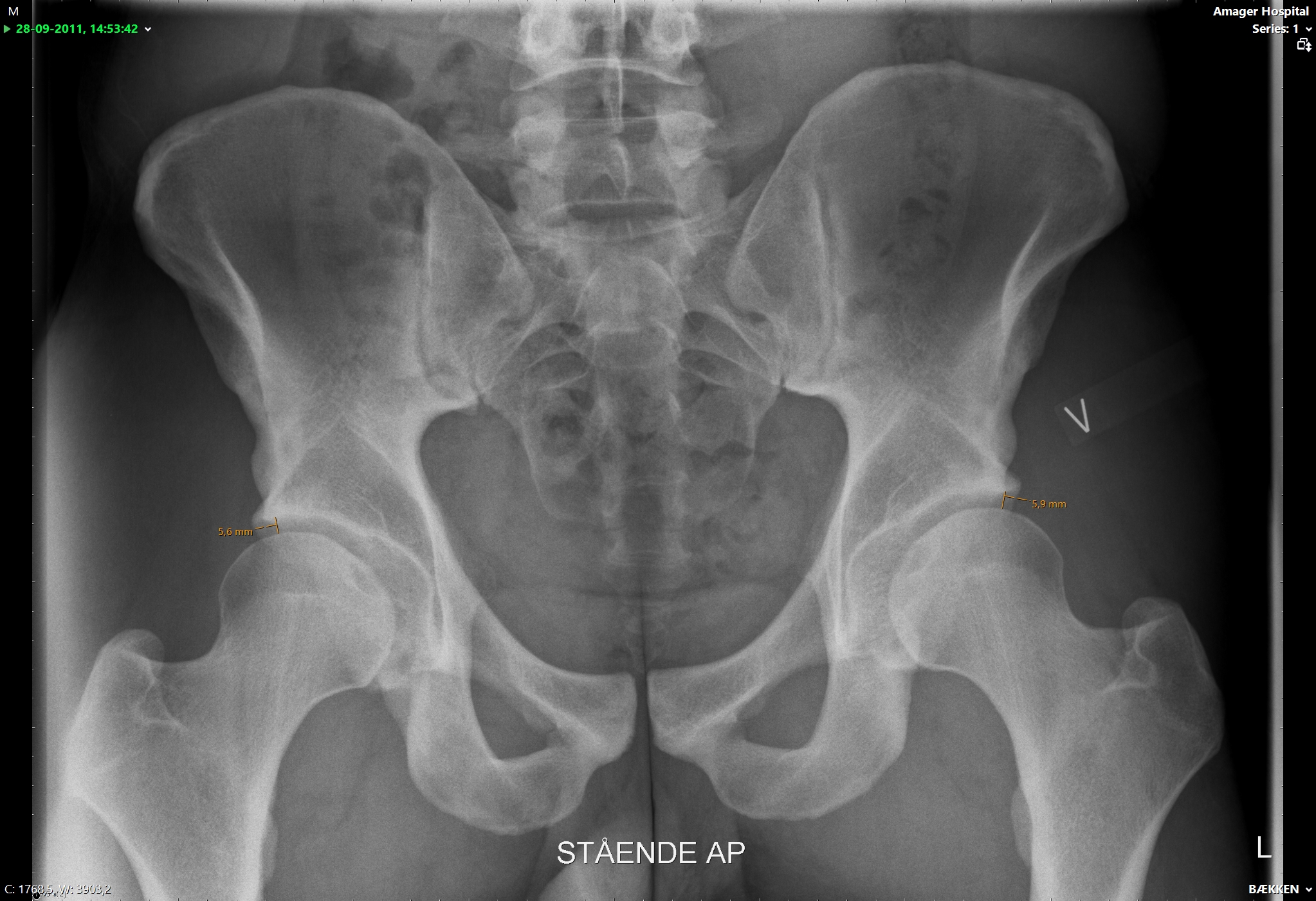


**Cross-over sign.**

**Definitions**: *Cross-over sign:* The anterior wall contour intersects and becomes lateral to the posterior wall contour. ^3^

**View**: Anteroposterior.

**Defines:** *Pincer morphology* as a positive Crossover sign AND posterior wall sign AND Ischial spine sign ^3^.


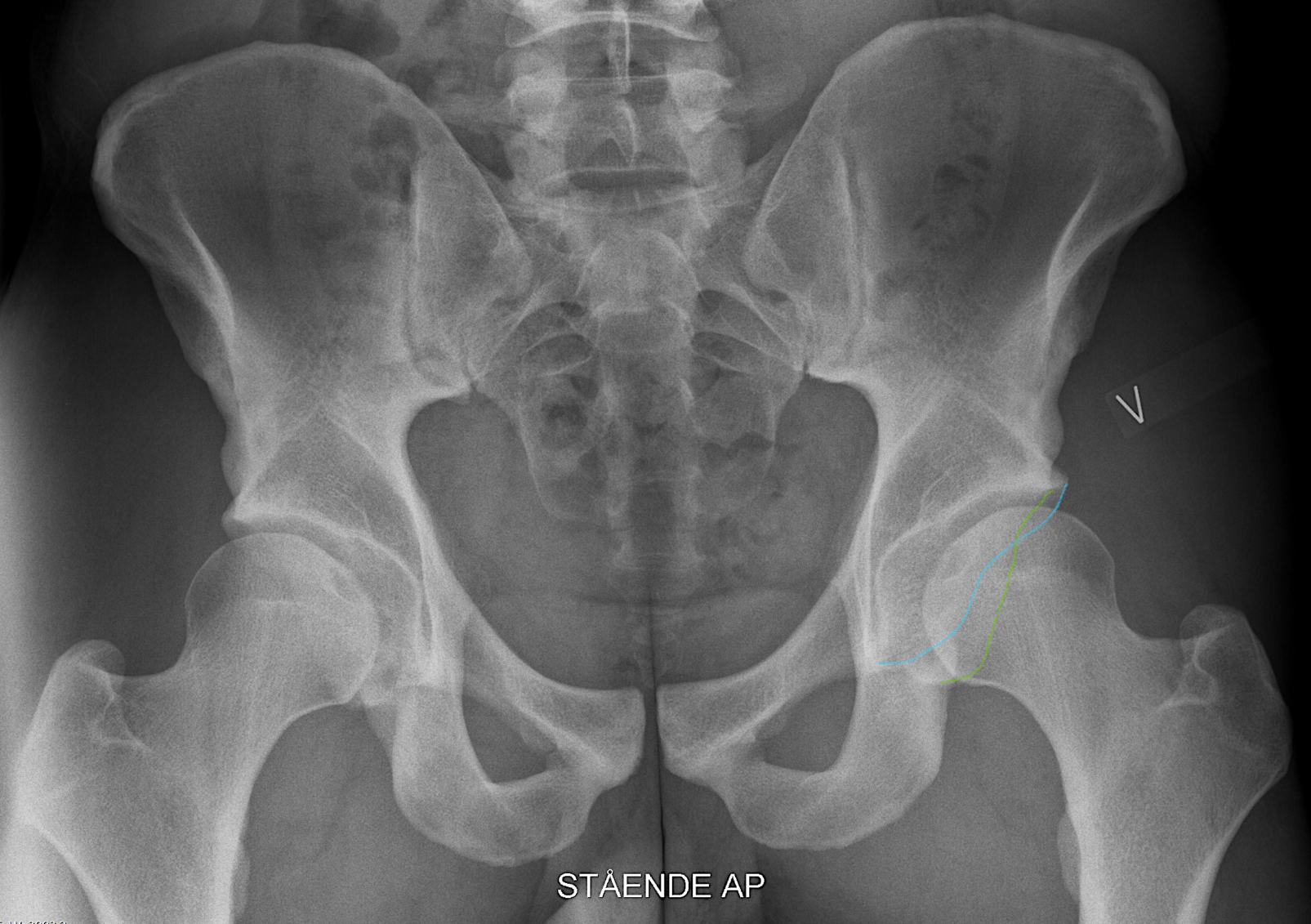


**Posterior Wall Sign.**

**Definition**: The posterior acetabular wall projection is medial to the femoral head center projection^3^.

**View**: Anteroposterior

**Defines:** *Pincer morphology* as a positive Crossover sign AND posterior wall sign AND Ischial spine sign ^3^.


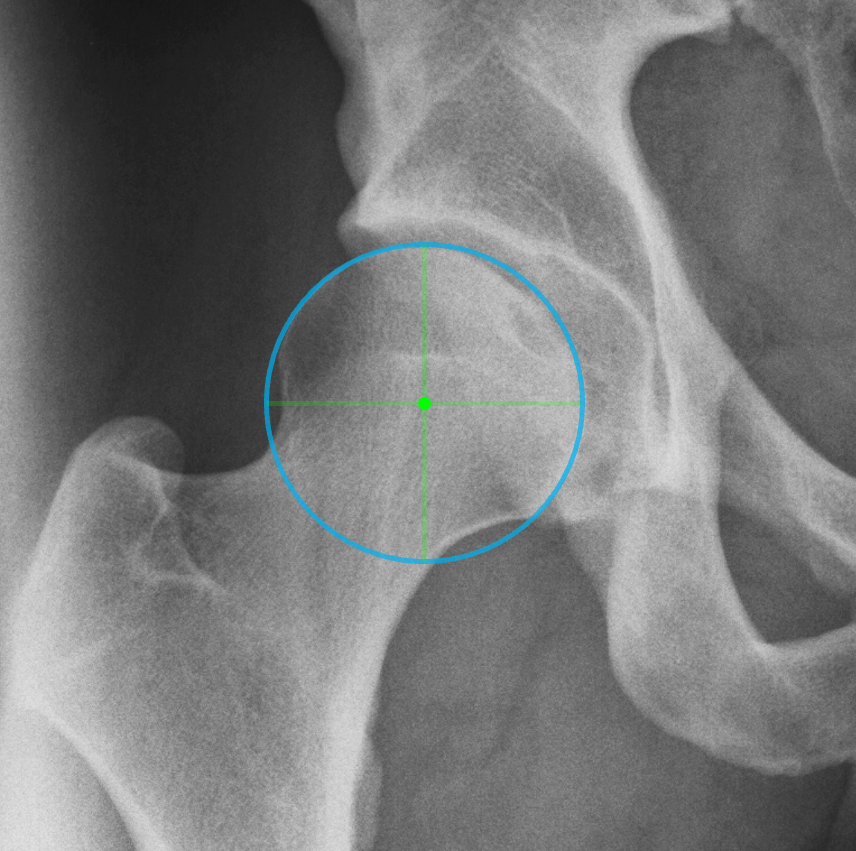


**Ischial Spine sign.**

**Definition:** The ischial spine is visible medially to the pelvic rim^3^. (just lateral to the green line on the image)

**View**: Anteroposterior.

**Defines:** *Pincer morphology* as a positive Crossover sign AND posterior wall sign AND Ischial spine sign ^3^.

**
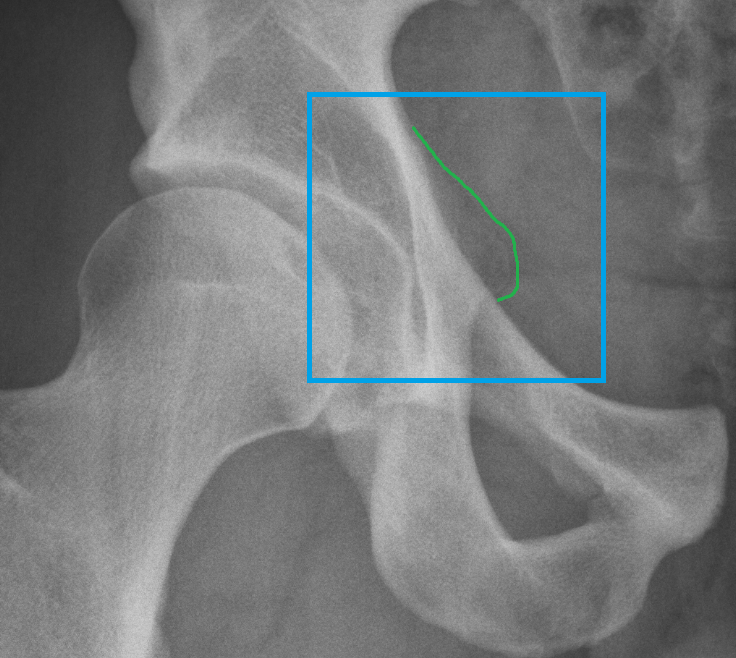
**

1. **The Copenhagen 5-Second Squeeze Test**

| 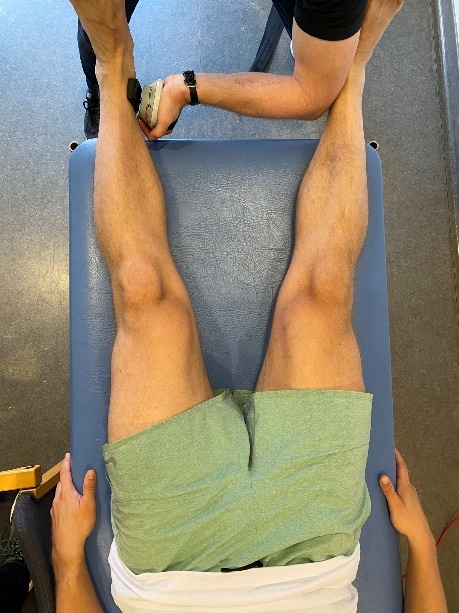 | 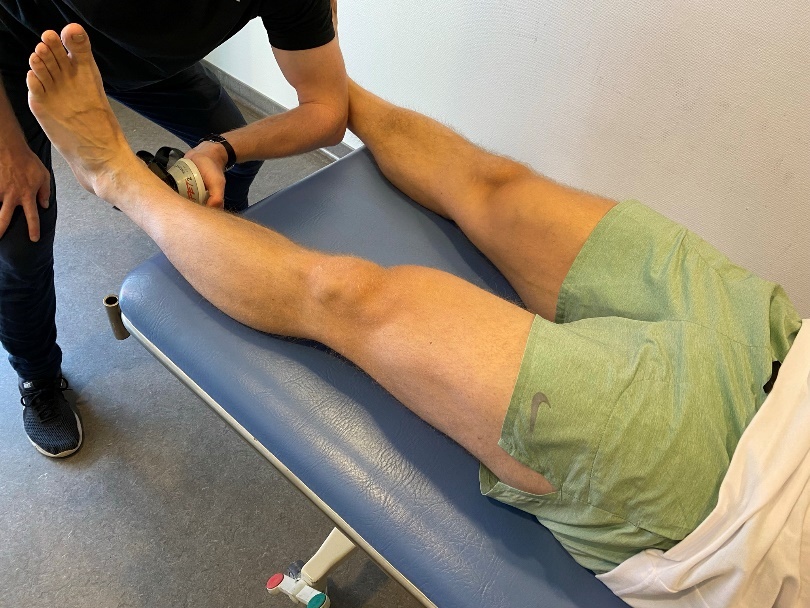 |
| --- | --- |

Reprinted from Physical Therapy Sport: *Fig. 1. The 5SST Setup in Nielsen MF, Thorborg K, Krommes K, et al. Hip adduction strength and provoked groin pain: A comparison of long-lever squeeze testing using the ForceFrame and the Copenhagen 5-Second-Squeeze test. Physical Therapy in Sport. 2022;55:28-36. doi:10.1016/j.ptsp.2022.02.002, license: CC BY 4.0.*

1. **Clinical examination and pain provocation tests**

| **Table S1 Pain provocation tests used for the clinical examination** | |
| --- | --- |
| **Pain provocation tests** | **Description of a positive test:** |
| Adductor longus palpation | Pain on palpation of the adductor longus muscle origin at the pubic bone |
| Gracilis palpation | Pain on palpation of the origin of the gracilis muscle at the pubic bone |
| Squeeze with 0° hip flexion† | Pain at the adductor insertion at the pubic bone on bilateral hip adduction in 0° hip flexion against manual resistance at the ankles |
| Passive adductors stretch | Pain on passive stretching of the adductor muscles |
| Psoas palpation (supra-inguinal) | Pain on palpation of the psoas muscle through the abdomen right above the inguinal ligament |
| Iliopsoas palpation (infra-inguinal) | Pain on palpation of the iliopsoas muscle just below the inguinal ligament, medial to the sartorius muscle, and lateral to the femoral artery |
| Passive hip extension in the modified Thomas test | Pain on passive stretching of the hip flexors in the modified Thomas test position |
| Resisted hip flexion in 90° hip and knee flexion | Pain on manually resisted single-leg hip flexion in 90° hip and knee flexion with the patient supine |
| Resisted hip flexion in the modified Thomas test | Pain on manually resisted single-leg hip flexion in the modified Thomas test position |
| Snapping hip test | Pain and snapping hip on active hip flexion to 90°, followed by active combined extension and abduction of the hip. |
| Rectus abdominis palpation ‡ | Pain on palpation of the rectus abdominis muscle at the pubic bone |
| Conjoint tendon palpation | Pain on palpation of the conjoint tendon at the pubic tubercle |
| Resisted sit-up ‡ | Pain in the groin when performing a sit-up against manual resistance |
| External inguinal ring palpation | Pain on palpation of the external inguinal ring |
| Valsalva & inguinal palpation | Pain on valsalva manoeuvre during palpation of the inguinal canal with invagination |
| Inguinal canal palpation | Pain on palpation of the inguinal canal with invagination |
| Symphysis palpation ‡ | Pain on palpation of the symphysis joint |
| Posterior impingement test | Pain in the groin on passive movement of a single leg in an extension, abduction, and external rotation |
| FADIR | Pain in the groin on a combined passive hip flexion adduction internal rotation |
| FABER | Pain in the groin on a combined passive hip flexion abduction external rotation |
| Apprehension test | Pain in the groin on passive movement of a single leg in an external rotation, while the hip is extended over the end of an examination table. The contralateral hip is hold by the subject in 90° flexion. |
| Log roll test | Pain in the groin on passive movement of a single leg from end-range external rotation to end-range internal rotation with the hip neutral |
| The provoked pain should correspond to the patient’s known and recognizable groin pain. † The test was performed once per athlete, but assessed pain in sides; ‡ The test was performed once per athlete | |

1. **Clinical entities of groin pain**

| **Table S2** The 2015 Doha agreement on terminology for clinical entities of groin pain. Reproduced from Weir et al.^6^ | |
| --- | --- |
| **Entity** | **Definition** |
| Adductor-related groin pain | Adductor tenderness† (a positive Adductor longus palpation or Gracilis palpation) AND pain on resisted adduction testing (a positive Squeeze test with 0° hip flexion) |
| Iliopsoas-related groin pain | Iliopsoas tenderness† (a positive Psoas (suprainguinal) palpation or Iliopsoas (infrainguinal) palpation) |
| Inguinal-related groin pain | Pain in the inguinal canal region AND tenderness* of the inguinal canal (a positive Conjoint tendon palpation or External inguinal ring palpation). No palpable inguinal hernia present. |
| Pubic-related groin pain | Tenderness† of the pubic symphysis and immediately adjacent bone (a positive Symphysis palpation) |
| Hip-related groin pain | Clinical suspicion of hip-joint being primary cause of pain based on history and/or examination |
| † Tenderness is defined as "discomfort or pain when the area is palpated, and the athlete recognizes this to be their specific injury pain".^6^ | |

**References**

1. Harris PA, Taylor R, Thielke R, Payne J, Gonzalez N, Conde JG. Research electronic data capture (REDCap)—A metadata-driven methodology and workflow process for providing translational research informatics support. *J Biomed Inform*. 2009;42(2):377-381. doi:10.1016/j.jbi.2008.08.010

2. Serner A, Arnaiz J, Mosler A, et al. Classifying radiographic changes of the pubic symphysis in male athletes: Development and reproducibility of a new scoring protocol. *Eur J Radiol*. 2021;134(109452):7. doi:https://doi.org/10.1016/j.ejrad.2020.109452

3. Mascarenhas VV, Castro MO, Rego PA, et al. The Lisbon Agreement on Femoroacetabular Impingement Imaging—part 1: overview. *Eur Radiol*. 2020;30(10):5281-5297. doi:10.1007/s00330-020-06822-9

4. Nepple JJ, Prather H, Trousdale RT, Clohisy JC, Beaulé PE, Glyn-Jones S. Diagnostic Imaging of Femoroacetabular Impingement. *J Am Acad Orthop Surg*. 2013;21:7.

5. Mygind-Klavsen B, Lund B, Nielsen TG, et al. Danish Hip Arthroscopy Registry: predictors of outcome in patients with femoroacetabular impingement (FAI). *Knee Surg Sports Traumatol Arthrosc*. Published online April 25, 2018. doi:10.1007/s00167-018-4941-3

6. Weir A, Brukner P, Delahunt E, et al. Doha agreement meeting on terminology and definitions in groin pain in athletes. *Br J Sports Med*. 2015;49(12):768-774. doi:10.1136/bjsports-2015-094869
